# Transcriptomic Analysis of Short/Branched-Chain Acyl-Coenzyme a Dehydrogenase Knocked Out bMECs Revealed Its Regulatory Effect on Lipid Metabolism

**DOI:** 10.3389/fvets.2021.744287

**Published:** 2021-09-07

**Authors:** Ping Jiang, Ambreen Iqbal, Mengyan Wang, Xiaohui Li, Xibi Fang, Haibin Yu, Zhihui Zhao

**Affiliations:** ^1^College of Coastal Agricultural Sciences, Guangdong Ocean University, Zhanjiang, China; ^2^College of Animal Science, Jilin University, Changchun, China

**Keywords:** ACADSB, CRISPR/Cas9, DEGs, lipid metabolism, fatty acid metabolism

## Abstract

The acyl-CoA dehydrogenase family of enzymes includes short/branched-chain acyl-CoA dehydrogenase (*ACADSB*), which catalyzes the dehydrogenation of acyl-CoA derivatives in fatty acid metabolism. Our previous findings suggested that *ACADSB* was a critical candidate gene affecting milk fat synthesis by comparing the transcriptome in bovine mammary epithelial cells (bMECs) from Chinese Holstein dairy cows producing high-fat and low-fat milk as well as gene functional validation studies on the cellular level. In the present study, *ACADSB* in bMECs was knocked out (KO) using a CRISPR/Cas9 system, and mRNA transcriptome was further sequenced to verify the function of the *ACADSB* gene and analyze its correlation with lipid metabolism. The findings revealed that 15,693 genes were expressed, 1,548 genes were differentially expressed genes (DEGs), and 6,098 GO terms were enriched, of which 637 GO terms were greatly enhanced, such as phospholipid-translocation ATPase activity (GO:0004012), lipoprotein lipase activity (GO:0004465), acyl-CoA desaturase activity (GO:0016215), and so on. The analysis by KEGG showed that DEGs were distributed over 247 pathogens, of which 49 were significantly enriched, including the metabolism of fatty acids (PATH: 01212), metabolism of glycerolipid (PATH: 00561), and signaling of adipocytokines (PATH: 04920). The CHOL, TGs and FFA contents in bMECs were reduced when the *ACADSB* gene was knocked out. The RT^2^ Profiler PCR array also revealed that the loss of the *ACADSB* gene changed the expression levels of functional genes involved in lipid metabolism, including *ACADL, ACOX2, ACAT2, and FABP3*. In conclusion, the current findings show that *ACADSB* is a key regulator of lipid metabolism in bMECs. The ACADSB^−/−^ bMECs could also be useful genetic material and tools for future research into gene functions related to lipid and fatty acid metabolism. It will be valuable for revealing the gene regulatory roles and molecular mechanisms in milk fat synthesis.

## Introduction

Milk fat is the most significant energy component in milk, and it plays a critical role in milk quality. Milk has a total fat content of about 3–5%. However, with the advancement of technology, the genetic mechanisms involved in milk fat synthesis are hitherto (1). The short/branched-chain acyl-CoA dehydrogenase (*ACADSB*) ENSBTAG00000018041) gene has two alternatively spliced transcripts and is found on the bovine chromosome 26:43134415-43184345. In bovine mammary epithelial cells (bMECs), *ACADSB* is a vital gene involved in lipid and fatty acid metabolism and has regulated triglyceride (TG) synthesis (2). Fat is oxidatively decomposed by lipase in lipid metabolism into triglycerides and free fatty acids and further decomposed into acetyl-CoA intrusive to the cycle of tricarboxylic acids (TCA), which involves glucose, lipid, and amino acids metabolism (3–5). The *ACADSB* gene also belongs to the ACAD mitochondrial family (6, 7). When a gene for a key enzyme in the mitochondria is mutated or deleted, it directly impacts the mitochondria's energy metabolism and the organism's aerobic respiration and glycolysis (8, 9). As a key functional gene in the lipid and fatty acid metabolism pathways, *ACADSB* could be an important link in these major metabolic networks.

Using gene overexpression and RNA interference (RNAi) approaches, the goal of this study was to learn more about the regulatory roles of the *ACADSB*, a candidate gene in bMECs involved in lipid and FA metabolism. However, the knock-out model of *ACADSB* gene cells remains less obvious in these studies (2). Therefore, the bMECs line has been chosen, which is capable of synthesizing and secreting milk *in vitro* and the mammary tissue of dairy livestock. Moreover, it can be used as a test model for cells to study gene function linked to the metabolism of milk lipids. The gene-editing technology CRISPR-Cas9 allows for the genetic modification of a wide range of cell and animal models. As a result, using CRISPR-Cas9 editing technology to create a Knock out cell line model further verifies the function of the target gene that regulates lipid metabolism and uses high-throughput RNA-seq to unravel the complex molecular mechanisms underlying various biological processes.

## Materials and Methods

### Preparation of Cells and Cell Culture

The experimental samples in this research were bMECs that had been stored in our laboratory. The bMECs were purified and cultured from a healthy dairy cow according to previous work performed in our laboratory by tissue nubble culture method (10). The cells were cultured in a basal medium containing Dulbecco's modified Eagle's medium and Nutrient Mixture F-12 (DMEM/F12, HyClone, Logan, USA) containing 10% FBS (fetal bovine serum, Tian hang, Zhejiang, China), 25 μL hydrocortisone (25 g/ml, Abcam, Cambridge, UK), and 25 μL insulin (0.25 EU/mg, Abcam, Cambridge (HyClone, Logan, USA).

### Constructing Knockout Vector ACADSB^–/–^

The Zhang lab's pSpCas9(BB)-2A-GFP (PX458, addgene 48318) vector was chosen as the vector backbone because it expresses Cas9 Streptococcus pyogenes with an invariant sgRNA scaffold and cloning sites for inserting the guide sequence. Two complementary sgRNA oligo DNA were synthesized, annealed to form double-stranded DNA in the presence of 10 × NEB annealing buffer, and cloned into the BbsI sites of the PX458 targeting vector, according to the location and specificity. Transient transfecting bMECs resulted in the knockout of the *ACADSB* gene fragment. After 24 h, the cells were collected using trypsin without EDTA solution, and the cells were screened using flow cytometry to see if they could express a green fluorescent protein. The single positive cells were cultured in 96-well plates containing 20% serum and 1% antibiotics. Seven days later, under a microscope, a small pile of cell pellets can be seen. After digesting the cells and continuing culturing, a large population of cells can be observed under the microscope 15 days later.

### PCR Identification and Sequencing

The genomic DNA was extracted from the cells, and PCR amplification was performed using the *ACADSB* gene detection primers. The detection primer sequence was: *ACADSB* F 5′-CGATACATACAGGCTTTTGGAAG-3′; *ACADSB* R 5′-AAGATGAGGGGCAATGGCT-3′. The edited cell lines were screened by PCR products sequencing.

### Sample Collection and cDNA Library Construction

Total RNA was extracted with standard chloroform extraction from *ACADSB*^−/−^ and negative control cells. DNase I (NEB, Beijing, China) was used to treat total RNA and Trizol reagent to extract it (Invitrogen, USA). The RNA quality was checked using the Bioanalyzer 2200 (Aligent)and then stored at −80°C. The cDNA libraries for each pooled RNA sample were created using the VAHTSTM Total RNA-seq (H/M/R) according to the manufacturer's instructions.

### RNA Sequencing Mapping

Pair-end read mapping is a technique for mapping reads that have been split into two halves. Before the mapping was read, clear readings of the raw readings were obtained by removing adapter sequences, readings of >5% ambiguous bases (N) and low-quality readings containing more than 20% bases with <20. The clean readings were then aligned with the target genome.

### Differentially Expressed Gene (DEG) Analysis in ACADSB^–/–^ bMECs

Based on the fragments per kilobase of exon per million fragments mapped (FPKM) values, the expression information from RNA-seq data for the *ACADSB*^−/−^ bMECs and wild-type (WT) bMECs was acquired. The DEGs were identified and filtered using the EB-Seq algorithm (11). P-value and FDR analysis were used to identify up-regulated or down-regulated DEGs after significant expression analysis (12).

### Gene Enrichment Analysis and Pathway Analysis

The analysis of gene ontology (GO) was conducted to elucidate the biological consequences of unique genes insignificant or representative target gene profiles (13). NCBI (http://www.ncbi.nlm.nih.gov/), UniProt (http://www.uniprot.org/), and the Gene Ontology (http://www.geneontology.org/) provided the GO annotations. The significant GO categories were identified using Fisher's exact test, and the *P*-values were corrected using the FDR method. Pathway analysis was used to identify the effective path of KEGG differential genes. Fisher's exact test was used to select the significant route, and *P*-value and FDR defined the threshold of significance (14). Corrected *p* < 0.05 has been considered to be significantly enhanced.

### Construction of the Interactive Network

KEGG refers to as we are all aware of several biological processes, including metabolism, membrane transport, signal translation and cell cycle pathways. The DEGs were enriched by biological pathways and the graphic representations of pathways using Cytoscape 3.6.1 (15). The study of gene interaction can reflect the function of genes and the regulatory role of genes.

### Analysis of ACADSB Expression by Quantitative Real-Time Polymerase Chain Reaction (qRT-PCR) and Western Blotting (WB) in the Knockout Cells

With the help of an RNA extraction kit, total RNA was extracted from the *ACADSB*^−/−^ and WT bMECs (Analytik Jena, Germany). A spectrophotometer was used for the concentration and purity of the RNA and further confirmed with 1.5 per cent of the agarose gel electrophoresis. The cDNA was obtained with a synthesis kit for cDNA (TaKaRa Biotechnology, RR047A, Dalian, China). The first printing was made of 5′-AGCTACTGTGCGCTGCTG-3′ and reverse priming, 5′-TCCATTGCTGTTGAG-3′. Then, the qRT-PCR was performed with SYBR® Premix Ex Taq TM (TaKaRa Biotechnology, RR420L, Dalian, China) (2). The following conditions were used for PCR amplification in a 10 μL reaction volume: an initial denaturing step of 95°C for 30 s, followed by 40 cycles of 95°C for 10 s, and then 60°C for 45 s. The concentrations of all the samples were adjusted to the same level. qRT-PCR was used to determine mRNA expression, with β-actin serving as an internal control gene. All qRT-PCRs were carried out three times in total.

The total protein was extracted from the *ACADSB*^−/−^ and WT bMECs using a radio immunoprecipitation assay buffer (RIPA, AR0105, BOSTER, China) containing protease and phosphatase inhibitors. The Enhanced BCA Protein Quantitation Assay Kit was used to determine the total protein concentration (KeyGEN BioTECH, KGP902, Jiangsu, China). Technical replicates were used for the three experiments on the same sample, and protein concentration was measured in triplicate. The rabbit polyclonal anti-*ACADSB* antibody (1:1550 dilution in 1 TBS, sigma Antibodies, AV-54586, China) and tubulin (1:20, 000 dilution in 1 TBS, bioss, bs-4511R, Beijing, China) were then incubated for 16 h at 4°C on the polyvinylidene fluoride membrane. A gray value analyzer was used to calculate the relative expression of the target proteins in different cells (Tanon 5200, Shanghai, China).

### Validation of mRNAs by RT^2^ Profiler PCR Array

An RNeasy Mini Kit extracted 1 μg of total RNA from the bMECs (Qiagen, 74134, Frankfurt, Germany). Then, according to the protocol, cDNA was synthesized using an RT^2^ First Strand Kit (Qiagen, 330404, Frankfurt, Germany). Mx3005p was used to perform the qRT-PCR (Agilent Stratagene, California, USA). An RT^2^ Profiler PCR Array was used to determine the transcript levels of genes involved in the lipid and fatty acid metabolism pathways (Qiagen, CLAB24070A, Frankfurt, Germany). 5 different housekeeping genes were selected as reference genes, β-actin (*ACTB*), glyceraldehyde-3-phosphate dehydrogenase (*GAPDH*), tyrosine 3-monooxygenase (*YWHAZ*), hypoxanthine phosphoribosyltransferase 1 (*HPRT1*) and TATA-box binding protein (*TBP*). The Ct values were standardized based on the gene selection. The expression fold change/regulation was calculated using the delta/delta Ct method of the data analysis web portal in which delta Ct is calculated between the gene of interest (GOI) and the average reference genes (HKG), followed by delta-delta Ct calculations. Then the fold change was calculated with formula 2 (^−delta delta Ct^) (5).

### Analysis of the Triglyceride, Cholesterol and Free Fatty Acids Contents in bMECs

TG detection kit was used to determine the content of TGs in the samples. The total CHOL content of cells was determined using a tissue/cell total CHOL assay kit (Applygen Technologies, Beijing, China). The FFA assay kit was used to determine the amount of free fatty acids in the sample (Solarbio life sciences, Beijing, China). WT and *ACADSB*^−/−^ cells were seeded at a density of 2 × 10^5^ cells per well in culture plates. The concentrations were measured using a microplate reader (YongChuang SM600, Shanghai, China) after the cellular contents of TGs, CHOL, and FFA were adjusted by each microgram of protein. The TGs, CHOL, and FFA of cells were extracted following the manufacturer's instructions.

### Statistical Analysis

Experimental data is displayed as ± SEM means. The *P*-value (*p* < 0.05) has been defined as statistically significant differences. A comparative Ct method (2^−delta delta Ct^) was used for qRT-PCR data on relative gene expression. GraphPad Prism 6 software (GraphPad, San Diego, CA, USA) is used in the data analysis with a two-tailed *t*-test. The online RT^2^ Profiler PCR Array data analysis software (https://geneglobe.qiagen.com/cn/analyze) analyzed the relative expression information for genes.

## Results

### Generation of ACADSB^–/–^ bMECs

**Figure 1:**
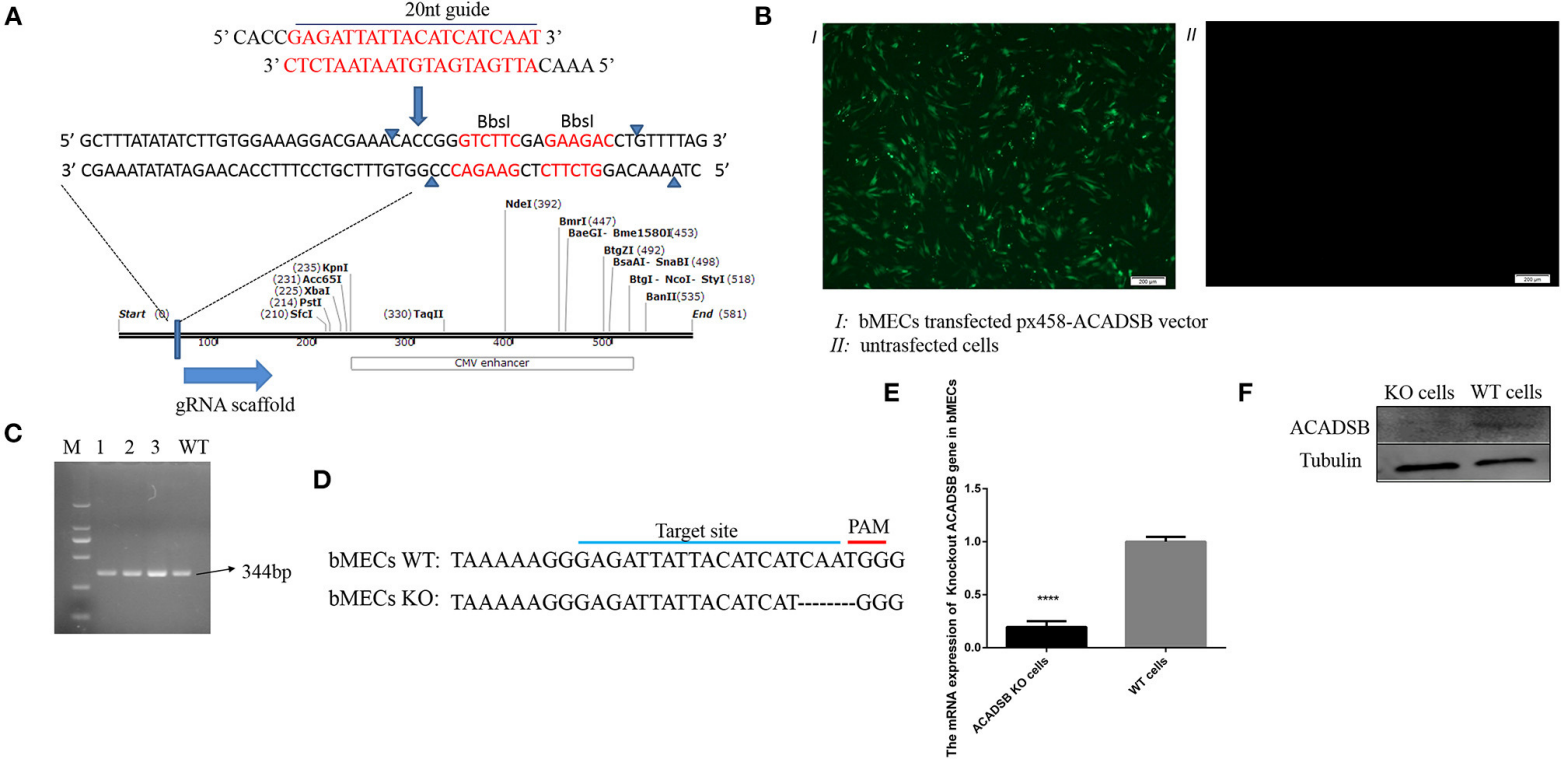
Construction of *ACADSB* gene knockout vector and identification of mRNA and protein levels. **(A)** Construction of *ACADSB* gene recombinant knockout vector (PX458-*ACADSB*). **(B)** PX458-*ACADSB* recombinant knockout vectors were transfected into bMECs. **(C)** The single positive cells were screened which can express green fluorescent protein by flowing cytometry and then were cultured for extracting the genome DNA to identify the edited cell line by PCR. **(D)** The sequence of the monoclonal PX458-*ACADSB* knockout cell line was compared with the wild cell line, the gRNA sequence was missing 4 bases near the PAM site, causing a frameshift mutation. **(E)** *ACADSB* relative mRNA expression levels in *ACADSB*^−/−^ bMECs and wild type bMECs line. **(F)** *ACADSB* relative protein expression levels in *ACADSB*^−/−^ bMECs and wild type bMECs line.

The exon 5, the conserved domain of the *ACADSB* gene opening read frame (ORF) sequence, was selected as a potential target sequence for *ACADSB*^−/−^ sgRNAs. BbsI restriction site behind the U6 promoter was recognized, and the enzyme ligated into the PX458 plasmid (Figure 1A). After the recombinant plasmid was transfected into bMECs through transiently transfecting, the cells containing *ACADSB* sgRNA were identified with green fluorescence under a fluorescence microscope (Figure 1B). One single live cell was screened by the flow cytometry and then transferred into 96-well plates for the culture of *ACADSB*^−/−^ bMECs. Upon agarose gel electrophoresis, WT cells demonstrated a 344bp band for the ACADSB PCR product (Figure 1C). In addition, the sequencing data of genomic DNA extracted from WT cells and *ACADSB*^−/−^ bMECs confirmed that the target sequence of *ACADSB* was effectively disrupted in bMECs (Figure 1D). The expression of *ACADSB*^−/−^ bMECs was determined by qPCR and WB technology. *ACADSB*^−/−^ bMECs showed a significant decrease compared with WT cells in the mRNA level (Figure 1E). *ACADSB*^−/−^ bMECs also showed a significant decrease compared with WT cells in protein level (Figure 1F).

### Analysis of the Triglyceride, Cholesterol and Free Fatty Acids Contents in bMECs

**Figure 2:**
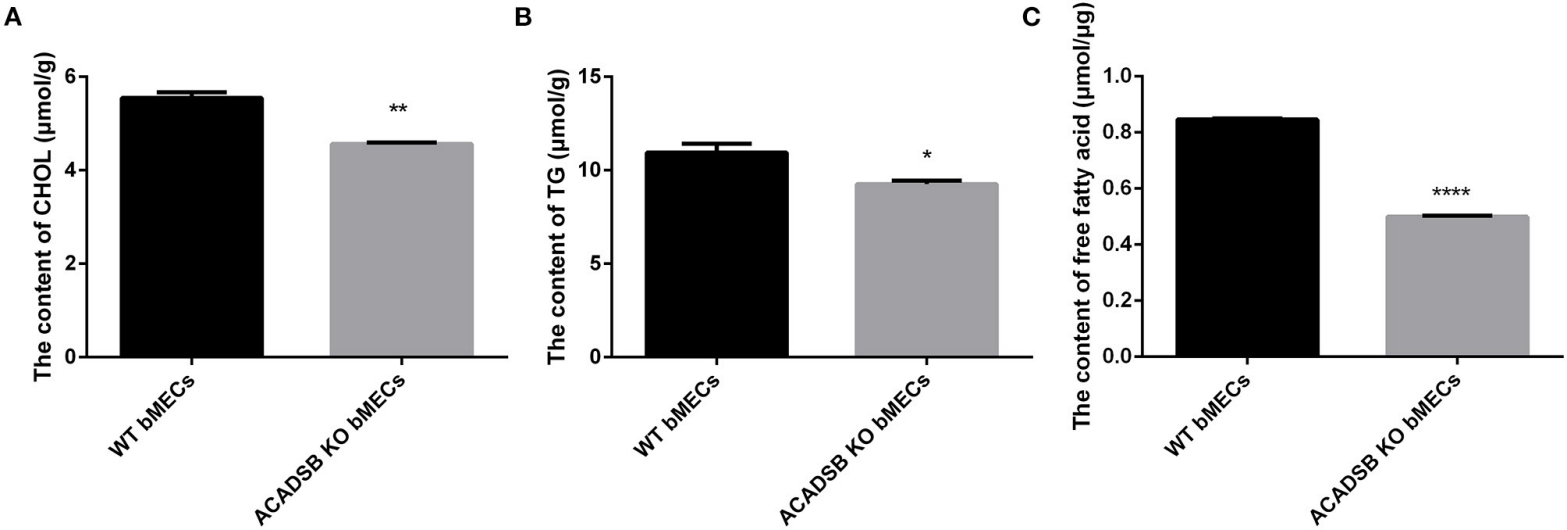
The effect of *ACADSB* on TGs, CHOL and FFA contents in bMECs. **(A)** The CHOL content in *ACADSB*^−/−^ bMECs was decreased compared with that in the WT bMECs. **(B)** The TG content was lower in *ACADSB*^−/−^ bMECs compared with that in the WT bMECs. **(C)** The content of FFA was also significant inhibited in *ACADSB*^−/−^ bMECs than in the WT bMECs (Experimental data are shown as mean ± SEM). ^****^p < 0.0001, ^**^p < 0.01, ^*^p < 0.05.

The results showed that the CHOL content in *ACADSB*^−/−^ bMECs was decreased compared with that in the WT bMECs (*p* < 0.05, Figure 2A). Furthermore, the TG content was lower in *ACADSB*^−/−^ bMECs than in the WT bMECs (*p* < 0.05, Figure 2B). The content of FFA was also significantly inhibited in *ACADSB*^−/−^ bMECs than in the WT bMECs (*p* < 0.05, Figure 2C).

### Identification of DEGs in ACADSB^–/–^ bMECs

**Figure 3:**
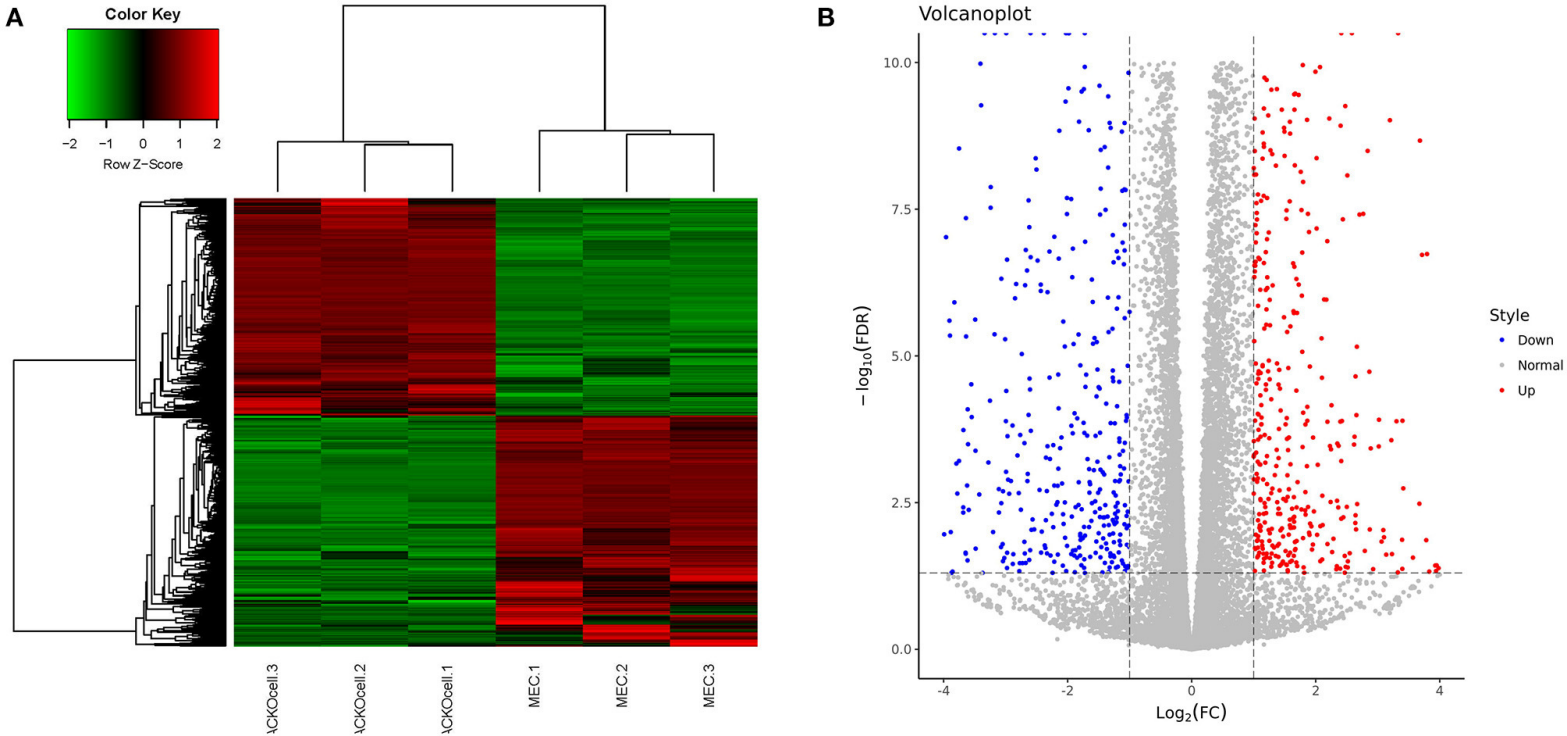
Identification of DEGs in WT and *ACADSB*^−/−^ bMECs by RNA-seq. **(A)** Hierarchical clustering heatmaps of WT and *ACADSB*^−/−^ bMECs. **(B)** Volcanoplot of WT and *ACADSB*^−/−^ bMECs.

RNA-seq analysis of *ACADSB*^−/−^bMECs was performed to clarify the mechanism of the *ACADSB* gene. A total of 1,548 DEGs were obtained for the *ACADSB*^−/−^ bMECs compared to the WT groups, including 750 upregulated DEGs, 798 downregulated DEGs, as shown in the hierarchical clustering heatmaps (Figures 3A,B) including five upregulated DEGs such as *ACADSB*, fatty acid desaturase 1*(FADS1)*, carnitine palmitoyltransferase 1C*(CPT1C)*, solute carrier family 27 member 5*(SLC27A5)*, 2,4-dienoyl-CoA reductase 2*(DECR2)*, and five downregulated DEGs such as protein kinase cAMP-activated catalytic subunit beta *(PRKACB)*, glycerol-3-phosphate dehydrogenase 2*(GPD2)*, acyl-CoA synthetase medium-chain family member 3*(ACSM3)*,acyl-CoA synthetase long-chain family member 1*(ACSL1)*, solute carrier family 27 member 6 *(SLC27A6)* showed in Table 1.

**Table 1:** The expression levels details of partial regulated genes in *ACADSB*^−/−^ *bMECs*.

**Gene ID**	**Log 2FC**	***P*-value**	**FDR**	**AC KO**	**MEC**	**KEGG ID**
*ACADSB*	−1.62	0.003	0.01	10.33	30.67	bta:504301
*FADS1*	−1.39	5.56E-09	3.24E-08	61.33	154	bta:533107
*CPT1C*	−1.21	3.11E-08	1.65E-07	77.33	172	bta:513710
*SLC27A5*	−0.82	0.19	0.28	12	20.33	bta:533016
*DECR2*	−0.53	9.1E-08	4.55E-07	488.33	680.33	bta:768256
*PRKACB*	0.78	0.005	0.01	83.67	46.67	bta:282323
*GPD2*	0.92	2.26E-20	3.42E-19	795.67	404.00	bta:504948
*ACSM3*	0.95	0.49	0.61	4.00	2.00	bta:533798
*ACSL1*	1.02	9.69E-24	1.79E-22	964.67	459.33	
*SLC27A6*	1.21	0.05	0.10	22.00	9.00	bta:537062

*FADS1, fatty acid desaturase 1; CPT1C, carnitine palmitoyltransferase 1C; SLC27A5, solute carrier family 27 member 5; DECR2, 2,4-dienoyl-CoA reductase 2; PRKACB, protein kinase cAMP-activated catalytic subunit beta; GPD2, glycerol-3-phosphate dehydrogenase 2; ACSM3, Acyl-coenzyme A synthetase ACSM3 mitochondrial; ACSL1, acyl-CoA synthetase long chain family member 1; SLC27A6, solute carrier family 27 member 6*.

### Validation of mRNAs in a Fatty Acid Pathway by RT2 Profiler PCR Array

To aid interpretation, the fold change results of relative genes involved in the lipid and fatty acid metabolism pathways are normalized in Table 2. *ACADSB*^−/−^ up-regulated seven genes, including acyl-CoA dehydrogenase long-chain (*ACADL*) and acyl-CoA oxidase 2 (*ACOX2*), according to the findings. *ACADSB*^−/−^ also caused the down-regulation of ten genes, including acetyl-CoA acetyltransferase 2 (*ACAT2*) and fatty acid-binding protein 3 (*FABP3*). The results were also consistent with the RNA-seq results, confirming the RNA seq's reliability.

**Table 2:** Fold changes in expression of genes involved in lipid and fatty acid metabolism that are regulated in *ACADSB*^−/−^ *bMECs*.

**Gene name**	**Fold change**	**Ref sequence**
*ACADL*	2.2377	NM_001076936
*ACADSB*	0.2998	NM_001017933
*ACAT2*	0.4544	NM_001075549
*ACOX2*	2.1317	NM_001102015
*ACSL1*	1.9889	NM_001076085
*ACSM3*	1.9212	NM_001035068
*BDH1*	0.5148	NM_001034600
*BDH2*	0.6338	NM_001034488
*CPT1B*	0.4095	NM_001034349
*CPT1C*	0.4095	XM_002695120
*DECR2*	0.6426	NM_001078122
*EHHADH*	1.7435	NM_001075780
*FABP3*	0.1858	NM_174313
*FADS1*	0.6079	XM_002699285
*GPD2*	1.6044	NM_001100296
*PRKACB*	1.6381	NM_174585
*SLC27A5*	0.4095	NM_001103273
*SLC27A6*	1.6155	NM_001101169

### Gene Ontology and Functional Enrichment Analysis in ACADSB^–/–^ bMECs

**Figure 4:**
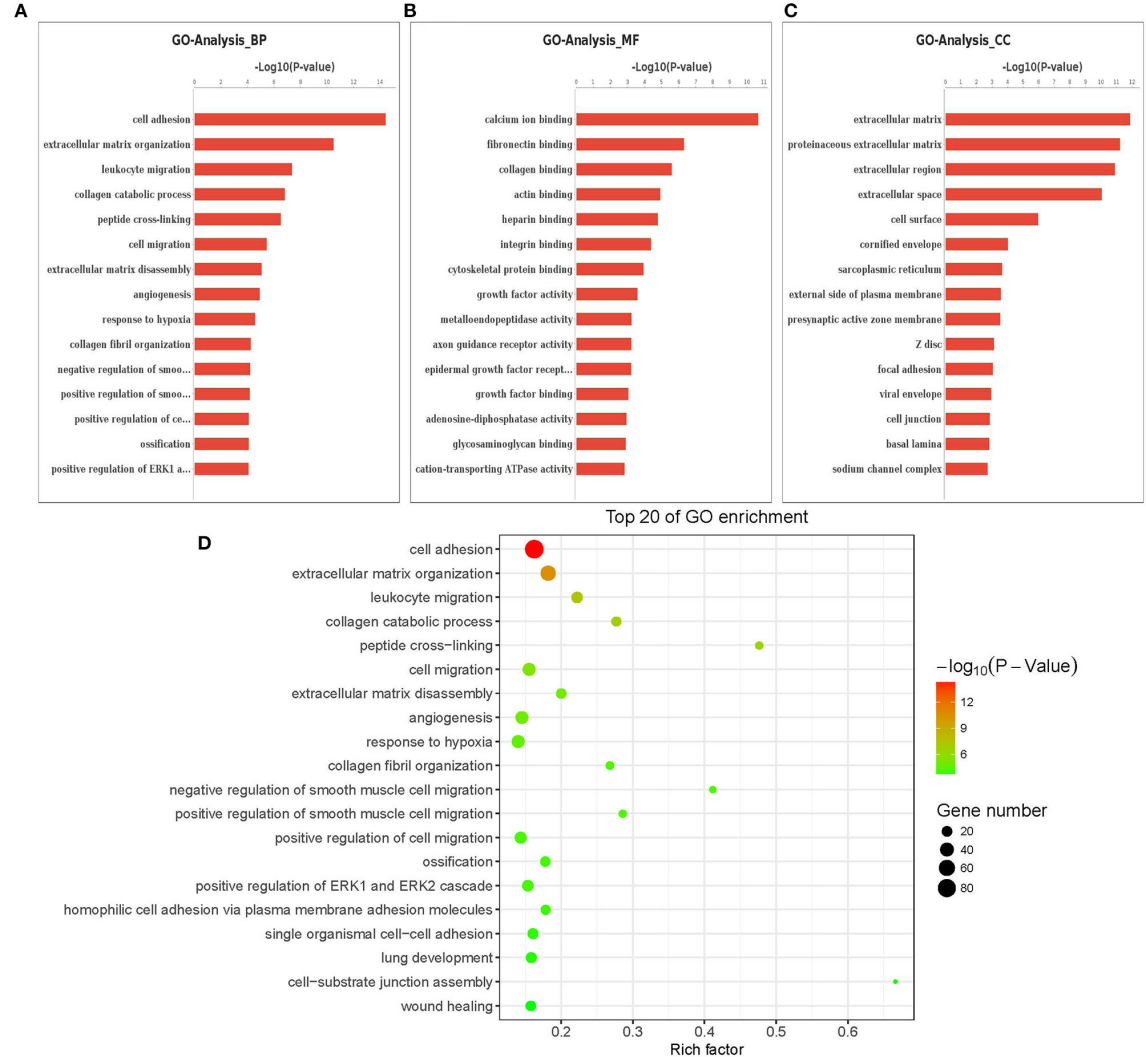
Enriched GO terms of DGEs in *ACADSB*^−/−^ bMECs. **(A)** The most significant differences in biological processes. **(B)** The most significant differences in cellular components. **(C)** The most significant differences in molecular functions. **(D)** The top 20 GO terms. GO, Gene Ontology. BP, biological process. CC, cellular component. MF, molecular function. DEG, differentially expressed gene.

**Figure 5:**
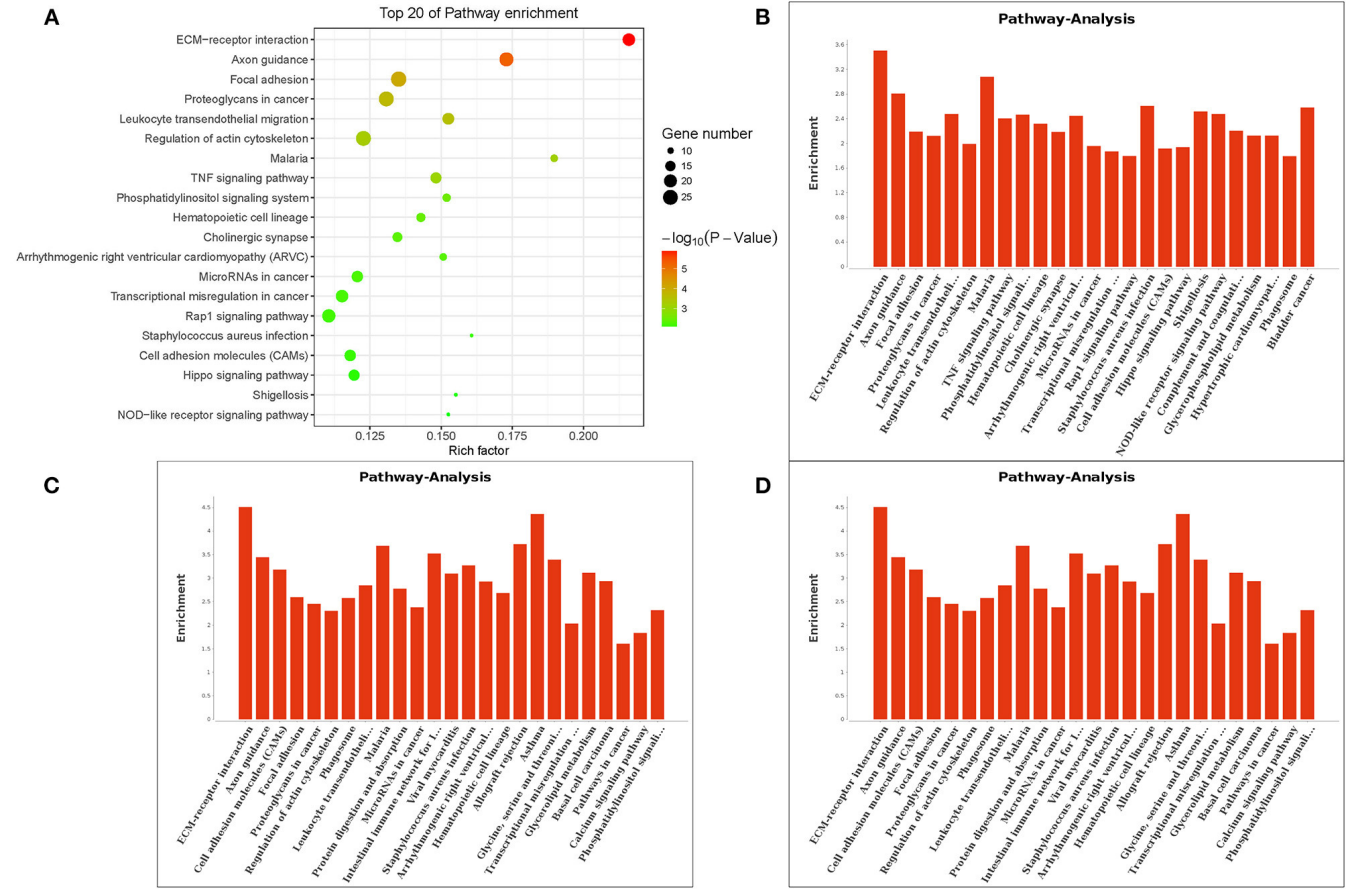
Enriched KEGG pathways of DGEs in *ACADSB*^−/−^ bMECs. **(A)** The top 20 enriched pathways for DEGs. **(B)** Pathway terms of DEGs. **(C)** Pathway terms of down-regulated genes. **(D)** Pathway terms of up-regulated genes. KEGG, Kyoto Encyclopedia of Genes and Genomes.

The Gene Ontology (GO) term functional enrichment demonstrates that DEGs have been enriched in 6,098 GOs, with 637 GOs significantly enriched, including 405 biological GO processes (Figure 4A), 113 molecular GO in Figure 4B and 45 cellular GO components (Figure 4C). Based on the molecular function, primary including ligand-activated sequence-specific DNA binding RNA polymerase II transcription factor activities (GO: 0004879), including insulin-like growth factor I binding (GO: 0031994). The biological process main including localization of cell adhesion (GO: 0007155) and extracellular matrix organization (GO: 0030198). Extracellular matrix (GO:031012) and proteinaceous extracellular matrix (GO: 0005578) are included in the cellular components. Figure 4D shows the top 20 GO terms, ordered by corrected P-value in ascending order. *ACADSB*^−/−^ bMECs have been subject to the enrichment analysis for KEGG pathways to explore the pathways associated with DEGs. Figure 5A juxtaposes the most significant pathway among the top 20 enriched pathways in the *ACADSB*^−/−^ bMECs group. In total, 247 pathways were discovered, with 49 pathway terms significantly enriched in Figure 5B. Down-regulated genes were significantly enriched in 27 pathways in *ACADSB*^−/−^ bMECs, with the ECM-receptor interaction (PATH: 04512) and glycerolipid metabolism (PATH: 00561) were significantly enriched in *ACADSB*^−/−^ bMECs, and ECM-receptor interaction pathway showed the highest enrichment in Figure 5C. Figure 5D shows that up-regulated genes are enriched in 46 pathways, including the Adipocytokine signaling pathway (PATH: 04920), Glycerophospholipid metabolism (PATH: 00564), and the Oxytocin signaling pathway (PATH: 04921).

**Figure 6:**
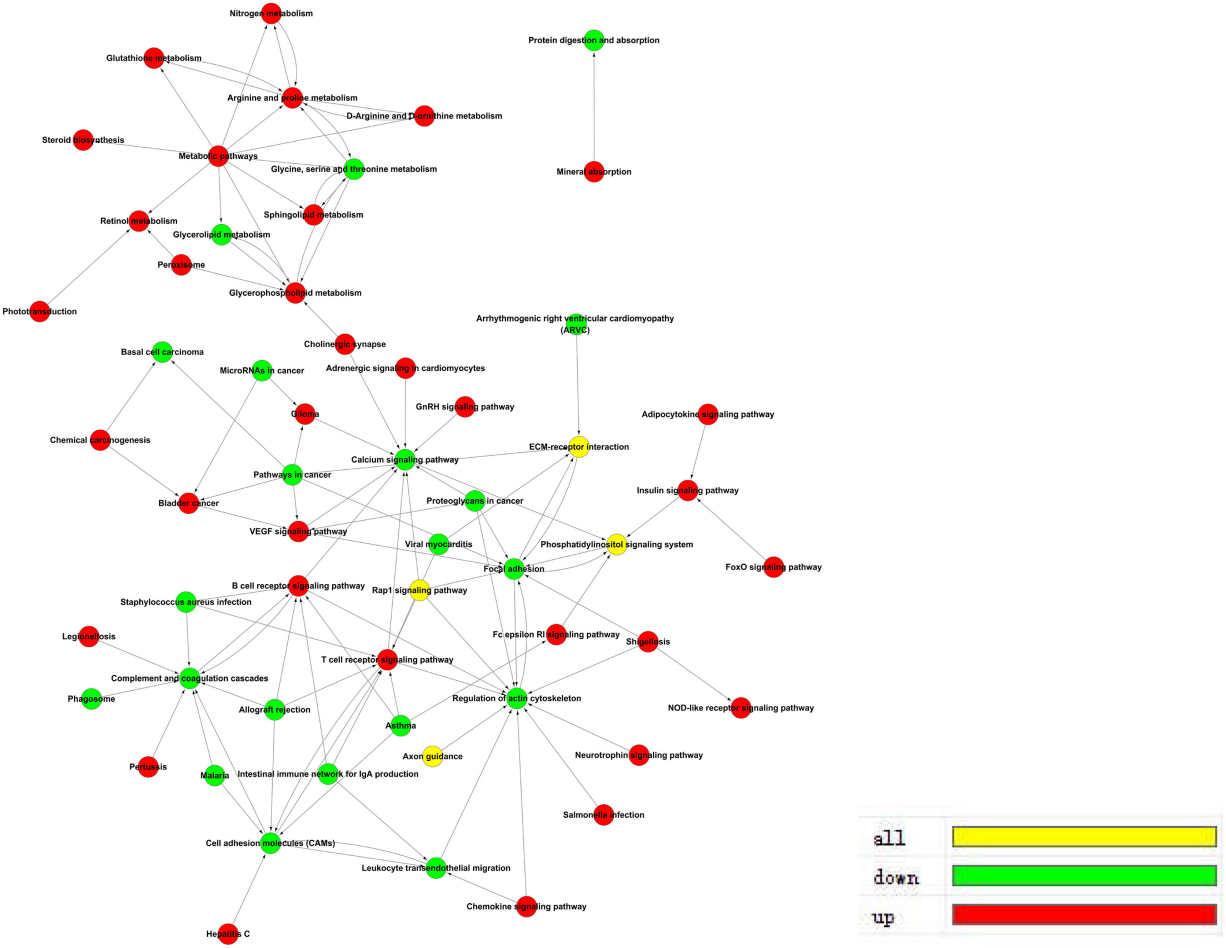
Pathway-Act network in *ACADSB*^−/−^ bMECs.

The pathway-act network represents the interaction of different pathways to explore interactions in an enriched biologic direction. The red dots represent up-regulated pathways, and the green dots represent down-regulated pathways shown in Figure 6.

### Predictive Analysis of Interaction Relationship Between Differential Genes

**Figure 7:**
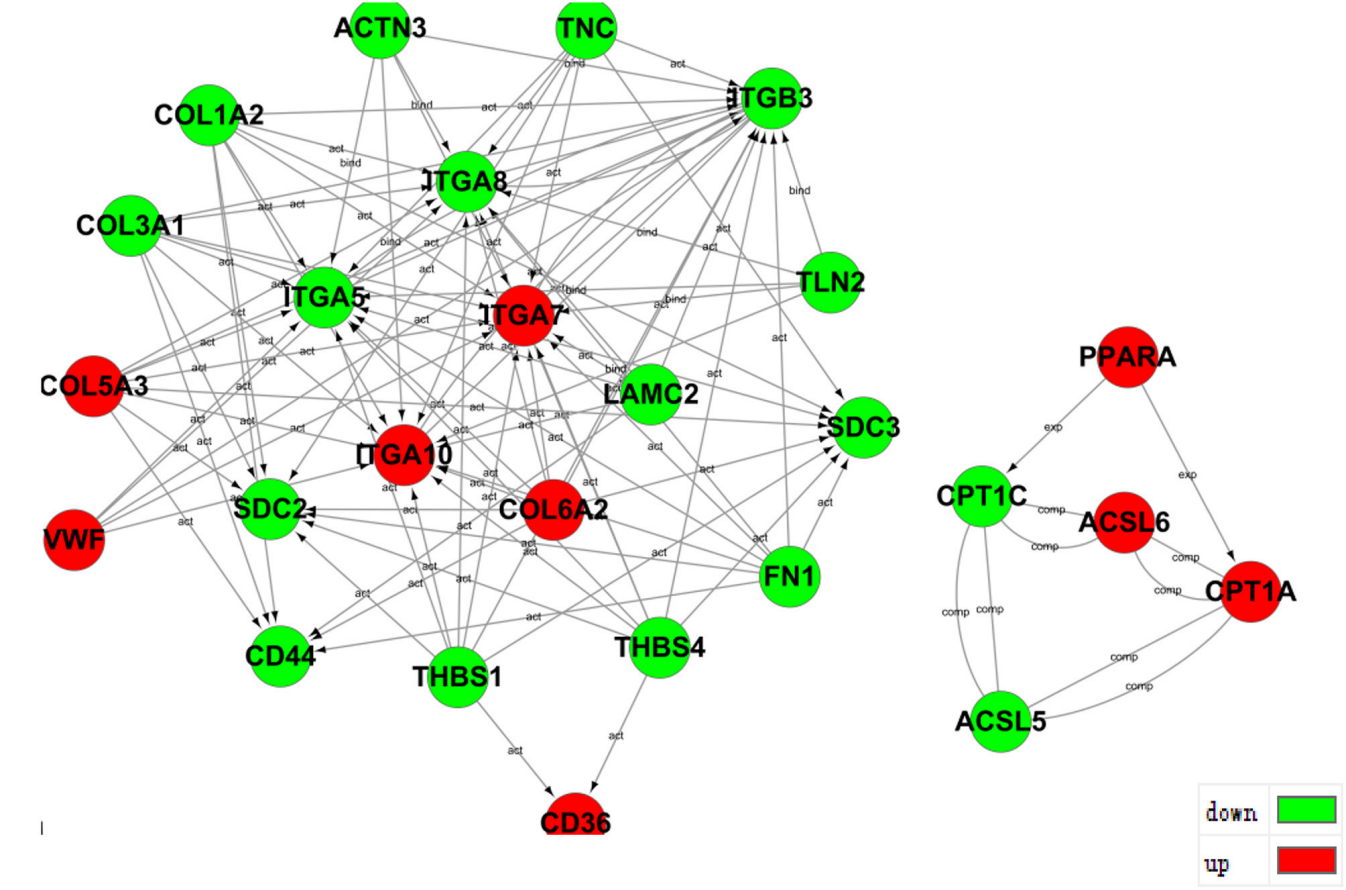
Prediction of protein interaction among partial differentially expressed genes.

The transcriptome analysis of differential expression genes protein interaction relationship in *ACADSB* gene knockout cells line revealed that up-regulated gene *PPARA* might promote the expression of up-regulated gene *CPT1A* and down-regulated gene *CPT1C*, which led to the up-regulated gene *CPT1A* and the down-regulated gene *CPT1C* may interact respectively with the up-regulated gene *ACSL6* and the down-regulated gene *ACSL5* to form a complex. The up-regulated genes *COL5A3* and *COL6A2* and the down-regulated genes *COL1A2, LAMC2, FN1*, and *COL3A1*, may collaborate to activate the down-regulated gene *CD44* (Figure 7), intimating that the *ACADSB* gene can influence *CD44* transcription levels by regulating the expression of other genes.

## Discussion

The term “gene-editing technology” refers to the process of altering an organism's genome artificially. The efficiency of gene editing has been dramatically improved due to the tremendous development of CRISPR/Cas9 technology. The effect of gene modification on a variety of cell models and animal models in several species has been realized (16, 17)^1^. CRISPR/Cas9 technology is effective in gene editing in eukaryotic cells in a growing number of studies. Gene-editing production, as a novel gene tool, can be used to investigate gene functions that aren't clear (18). Using the CRISPR/Cas9 system, some researchers successfully constructed myostatin (*MSTN*) KO animals such as cattle, pigs, sheep, mice, and rabbits, which is a reliable and effective animal model for the study of muscle development and related diseases, as well as having a good application prospect in transgenic animal production by improving muscle quality (19–22)^2^. According to recent research, CRISPR/Cas9 technology was used to knock out or knock down various functional genes, resulting in the regulation of related metabolic pathways. The role of genes was clearly explained *in vivo* and in animal models. The variety of cell models also were widely used in molecular research or related diseases, such as Fragile X Mental Retardation 1 (*FMR1*) homozygous knock out human embryonic stem cell line, which can further study the biological importance of Fragile X Mental Retardation Protein(*FMRP*) at a cellular level (23); Regulator of G protein signaling 18 (*RGS18*) knockout cell line from a human embryonic stem cell line was constructed, which can further understand roles of RGS18 in biological and cellular processes (24); The diacylglycerol kinase θ (*DGK*θ) gene was knocked out in liver cancer cell line HepG2 to investigate the role of *DGK*θ in lipid metabolism and insulin resistance (25), etc. This study revealed that knocking out a target gene can help researchers learn much about how it plays a role in the various metabolic, pathway, and biological processes at the cellular level. As a result, bMECs can be used as an experimental model to investigate the function of *ACADSB*^−/−^, which can be used to validate whether *ACADSB*^−/−^ can regulate the synthesis of fat milk components and play a significant role in milk fat synthesis.

In this study, gRNA sequences were designed in the conserved regions of the candidate genes. The coding regions of the candidate genes were precisely targeted, solving the problem of RNAi technology's low interference performance.

The *ACADSB* gene, involved in fat milk synthesis, has previously been differentially expressed (2). The mammary gland tissue of dairy cows can be used as a bioreactor for most biological research because it has the physiological functions of synthesizing and secreting milk. However, there are some limitations to using this model to research TG synthesis in the mammary gland (26). Culturing *in-vitro* bovine breast epithelial cells can maintain this ability to synthesize and secrete milk. And bMECs can be used as cell models to study the connection between objective genes and a milk fat synthesis pathway. Therefore, we are developing the knockout *ACADSB* vector to construct a knockout *ACADSB* cell line, so we can continue exploring in-depth. The current study has shown that *ACADSB*^−/−^ may impact the CHOL, FAs, and TGs content of bMECs following past research (2). As anticipated, *ACADSB*^−/−^ cells contained a significantly lower content of triglycerides, cholesterol, and FAs than control cells, indicating that the *ACADSB*^−/−^ cell line was the potential and valuable tool to evaluate the expression of relatively lipid-associated genes.

GO Term enrichment study demonstrated that DEGs have been massively enriched by GO terms linked to lipid metabolism and GO terms associated with cell metabolism. The level of expression considerably up-regulated gene enrichment for the GO-Term Gene in a cell-line of *ACADSB*^−/−^and wild-type bMECs has shown DEGs like the CD36 molecule(*CD36*), a Low- Density lipoprotein receptor-related protein 1B(*LRP1B*), which are part of low-density lipoprotein receptor activity Go term(GO:0005041), CD44 molecule(*CD44*) gene involved in cytokine receptor activity in Go term(GO:0004896), insulin-like growth factor-binding protein 5(*IGFBP5*) involved in insulin-like growth factor II binding GO term (GO: 0031995). Furthermore, the GO term-mRNA network study aims at finding genes associated with multiple lipid metabolism-related GO terms, such as apolipoprotein B mRNA editing enzyme, catalytic polypeptide 1(*APOBEC1*), NPC1-like 1(*NPC1L1*), oxidized low-density lipoprotein (lectin-like) receptor 1(*OLR1*), peroxisome proliferator-activated receptor-alpha (*PPARA*), apolipoprotein L domain containing 1 (*APOLD1*), etc. Furthermore, the level of gene expression was significantly reduced. The relative expression of target genes involved in lipid metabolism or cell metabolism was also regulated by knocking out the *ACADSB* gene, according to GO term enrichment.

The KEGG enrichment analysis of differentially expressed genes in *ACADSB*^−/−^ cell line and wild-type bMECs revealed that 247 pathways were enriched in differentially expressed genes, 49 of which were significantly enriched (*p* < 0.05), and 27 pathways were enriched in down-regulated genes, such as ECM-receptor interaction (PATH:04512), glyceride metabolism pathway (PATH:00561), and phosphatidylinositol metabolism pathway (PATH:04070), protein digestion and absorption (PATH:04974), etc. Up-regulated genes are found in 46 pathways, including the glycerophospholipid signaling pathway (PATH:00564), the oxytocin signaling pathway (PATH:04921), the adipocytokine signaling pathway (PATH:04920), the insulin signaling pathway (PATH:04910), and the metabolic pathway (PATH:01100), among others. According to the research results, the complete loss of the *ACADSB* gene resulted in differences in the expression levels of genes involved in fatty acid and lipid metabolism, regulating the lipid metabolism process in bMECs.

Following the KEGG enrichment and GO term enrichment analysis, which indicated *ACADSB* gene involved in the lipid metabolism and fatty acids metabolism, therefore, relative genes which involve in the lipid and fatty acids pathway were selected to confirmed by qPCR array. The *ACADSB*^−/−^ gene qPCR array results revealed that the following genes were up-regulated: *ACADL, ACOX2, ACSL1, ACSM3, EHHADH, GPD2, PRKACB, SLC27A5, SLC27A6*, while the genes such as *ACAT2, BDH1, BDH2, CPT1B, CPT1C, DECR2, FABP3* were down-regulated, which is consistent with the *ACADSB*^−/−^ transcriptome sequencing results. The above findings suggest that knocking out the *ACADSB* gene may affect the differential candidate genes identified by RNA-seq, which are functional genes that influence lipid and fatty acid metabolism.

Predictive analysis in *ACADSB*^−/−^ cell lines for differential gene-protein interactions showed that *CD44* was activated by other multiple genes, and *CD44* expression is down-regulated, suggesting that the silence of the *ACADSB* gene leads to decreased CD44-expression regulation. In the previous study, *CD44* gene silencing has also been found to decrease the level of expression of the *ACADSB* gene. This shows that *ACADSB* and *CD44* genes may be able to interact, and their function both focuses on lipid metabolism and fatty acids metabolism. However, the expression levels of all genes are not consistent due to differently regulated genes. This also reminds us that in the future, the interactive relationship between two or several genes can also be a hotspot for further exploration, and the *ACADSB* and *CD44* genes may have a common effect on lipid metabolism, or one of the *ACADSB* and *CD44* gene may affect lipid metabolism by regulating the expression of another gene.

In conclusion, our findings revealed that *ACADSB* regulates the metabolism of FFAs, TGs, and CHOL by controlling the expression of related genes involved in the lipid metabolism pathway. The findings also showed that *ACADSB* is a pivotal gene that regulates related genes such as *ACADL* and *ACOX2*, playing a crucial role in lipid metabolism. This study discovered the role of the *ACADSB* gene in lipid metabolism in bMECs *in vitro* and identified several key players in the *ACADSB* gene's regulatory network in lipid metabolism, laying the groundwork for future research into the molecular mechanism by which *ACADSB* influences the synthesis of milk fat component *in vivo*. Furthermore, in this study, the *ACADSB*^−/−^ cell line model was established, which could be used as genetic material and directly applied to lipid metabolism-related gene molecular research.

## Data Availability Statement

The datasets presented in this study can be found in online repositories. The names of the repository/repositories and accession number(s) can be found below: NCBI GEO, accession no: GSE181131.

## Author Contributions

ZZ and PJ designed the study. XL and XF collected the samples. MW and HY collected the data. PJ conducted the bioinformatics analyses and wrote the manuscript. AI and PJ revised the manuscript. All authors read and approved the final manuscript.

## Funding

This work was supported by the youth Project of Innovative Strong School Engineering by the Department of Education of Guangdong Province (2019KQNCX042), the National Natural Science Foundation of China (No. 32002165, 31772562, and 32072717), and the Key Platform Project of Innovative Strong School Engineering by the Department of Education of Guangdong Province (2018302).

## Conflict of Interest

The authors declare that the research was conducted in the absence of any commercial or financial relationships that could be construed as a potential conflict of interest.

## Publisher's Note

All claims expressed in this article are solely those of the authors and do not necessarily represent those of their affiliated organizations, or those of the publisher, the editors and the reviewers. Any product that may be evaluated in this article, or claim that may be made by its manufacturer, is not guaranteed or endorsed by the publisher.

## Footnotes

^1^Salimi M, Mehravar M, Shirazi A, Sarvari A, Behzadi B. Epigenetic events and germ line transmission in rag1 knock-out chimeric mice produced by CRISPR/Cas9 technology. (2021). 10.21203/rs.3.rs-153876/v1 (Unpublished preprint).

^2^Zhang Y, Zheng Y, Wu L, Li Z, Cui K. Generation of heritable double muscle buttocks rabbits via myostatin mutation with CRISPR/Cas9 system. (2020). 10.21203/rs.3.rs-125471/v1 (Unpublished preprint).
