# Supplementary material for: Transcriptomic Analysis of Short/Branched-Chain Acyl-Coenzyme a Dehydrogenase Knocked Out bMECs Revealed Its Regulatory Effect on Lipid Metabolism
Source: Front Vet Sci. 2021 Sep 7;8:744287. doi: 10.3389/fvets.2021.744287 (PMC8453006; doi:10.3389/fvets.2021.744287)
Supplement: Supplementary file 1 [file Data_Sheet_1.ZIP › Dr Jiang original data 1/figure 1/ACADSB KO mRNA expression/ACADSB检测201-1Print Primer.pdf]

Primer Analysis Report

Sequence: NewSequence

```
(Sense primer)       5' CGATACATACAGGCTTTTGGAAG 3'
                        |||||||||||||||||||||||
             3' (38) GCTATGTATGTCCGAAAACCTTC (60) 5'

(Anti-sense primer)  5' AAGATGAGGGGCAATGGCT 3'
                        |||||||||||||||||||
            3' (381) TTCTACTCCCCGTTACCGA (363) 5'
```

Properties:

	Rating	Seq No	Length	Tm [° C]	GC%	ΔG [kcals/mol]	Activity [μg/OD]	Degeneracy	Ta Opt [° C]
Sense	92	38	23	58.9	43.5	-42.0	31.3	1	--
Anti-sense	100	381	19	59.2	52.6	-39.2	30.8	1	--
Product	90	--	344	84.4	39.8	--	--	--	51.8

Secondary structures of sense primer:

Most stable hairpin:

ΔG =-0.0 [kcals/mol] (3' Hairpin)

```
┌TTTTCGGACATACATAGC 5'
|   |||
└GGAAG 3'
```

Most stable dimer:

ΔG =-4.5 [kcals/mol] (3' Dimer)

```
5' CGATACATACAGGCTTTTGGAAG 3'
                |||     |||
             3' GAAGGTTTTCGGACATACATAGC 5'
```

No false priming sites found

Secondary structures of anti-sense primer:

No hairpins found

No dimers found

No false priming sites found

Secondary structures of primer pair:

Most stable cross dimer:

ΔG =-4.4 [kcals/mol] (3' Cross dimer)

```
5' CGATACATACAGGCTTTTGGAAG 3'
      |  |||       ||   |
   3' TCGGTAACGGGGAGTAGAA 5'
```
