# Supplementary figures and images for: Transcriptomic Analysis of Short/Branched-Chain Acyl-Coenzyme a Dehydrogenase Knocked Out bMECs Revealed Its Regulatory Effect on Lipid Metabolism
Source: Front Vet Sci. 2021 Sep 7;8:744287. doi: 10.3389/fvets.2021.744287 (PMC8453006; doi:10.3389/fvets.2021.744287)

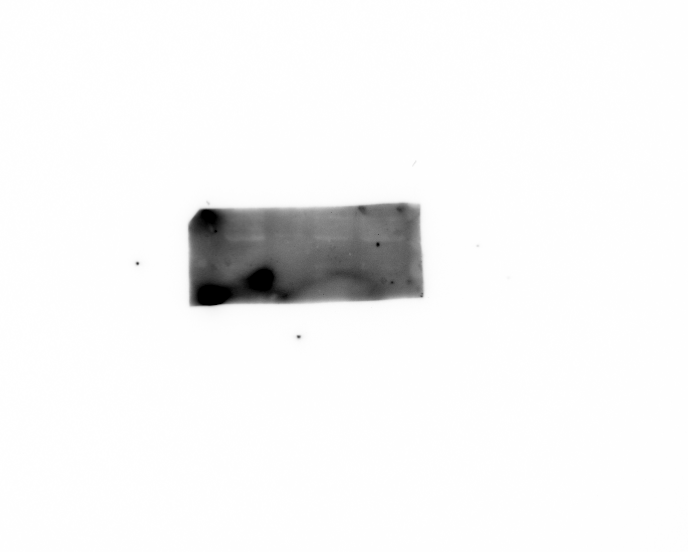

Supplement: Supplementary file 1 [file Data_Sheet_1.ZIP › Dr Jiang original data 1/figure 1/gel image/10.tif]

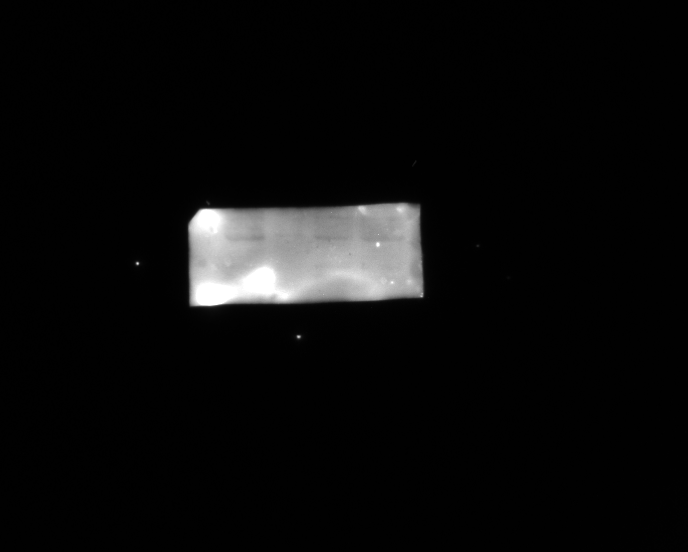

Supplement: Supplementary file 1 [file Data_Sheet_1.ZIP › Dr Jiang original data 1/figure 1/gel image/13.tif]

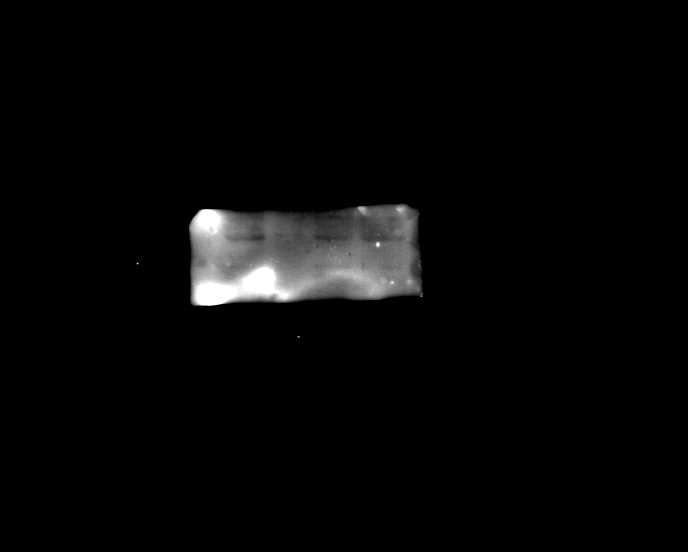

Supplement: Supplementary file 1 [file Data_Sheet_1.ZIP › Dr Jiang original data 1/figure 1/gel image/14.tif]

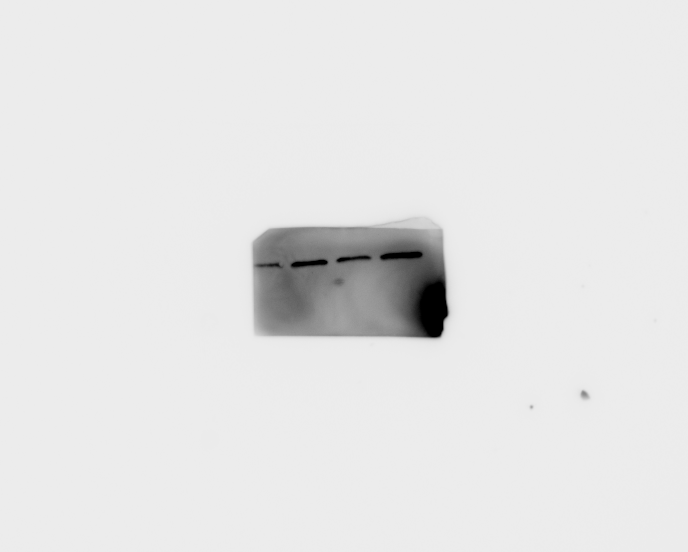

Supplement: Supplementary file 1 [file Data_Sheet_1.ZIP › Dr Jiang original data 1/figure 1/gel image/20.tif]

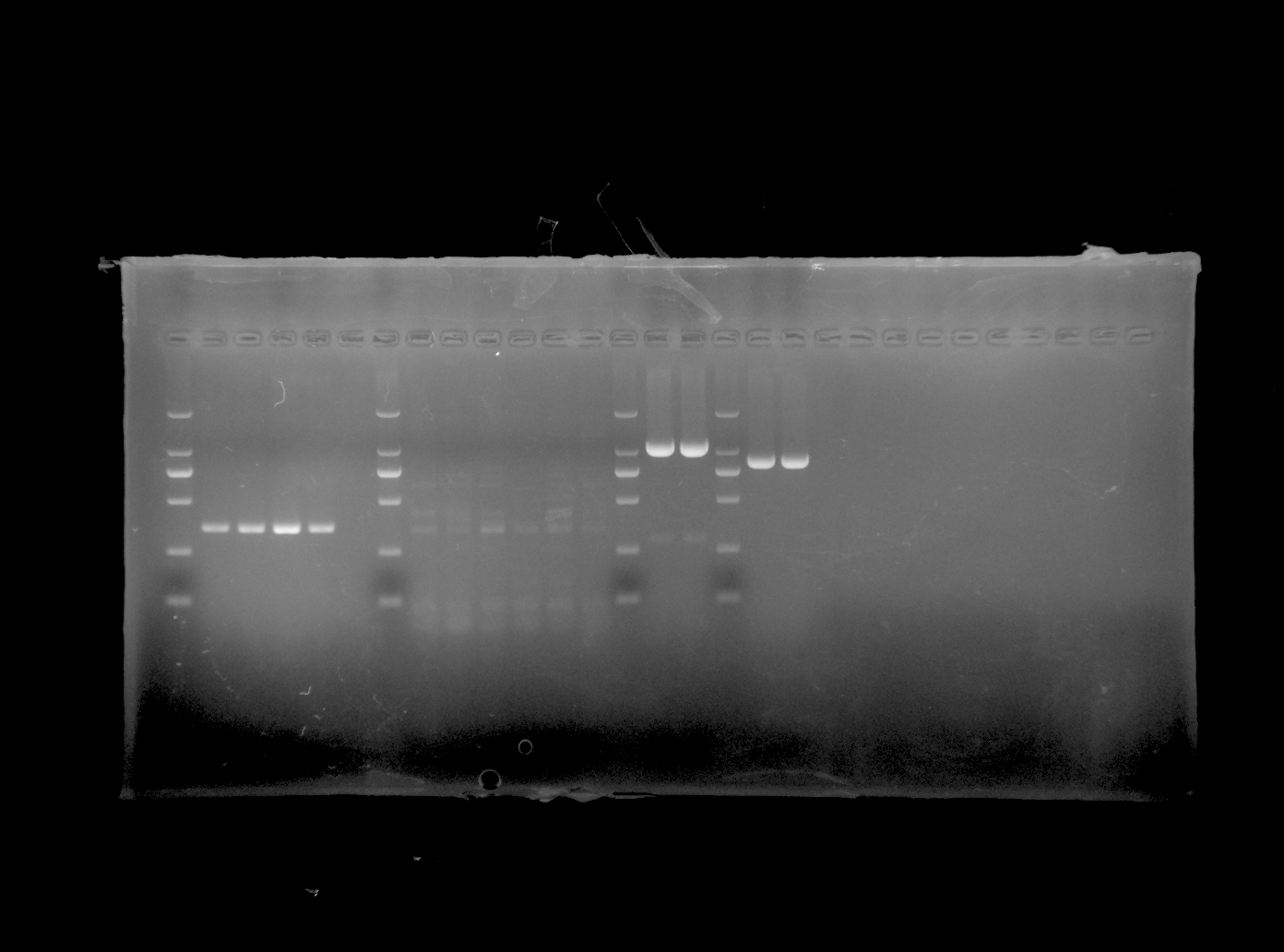

Supplement: Supplementary file 1 [file Data_Sheet_1.ZIP › Dr Jiang original data 1/figure 1/gel image/2018-1-17 CD44+ACAD的DNA pcr snp1+snp2.jpg]

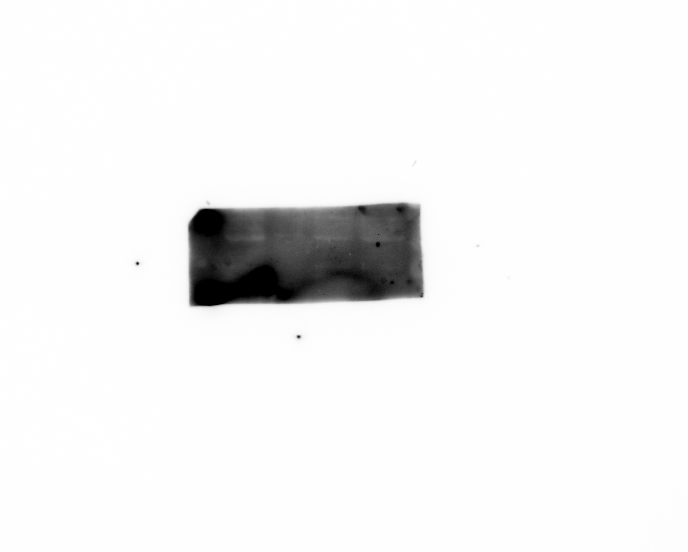

Supplement: Supplementary file 1 [file Data_Sheet_1.ZIP › Dr Jiang original data 1/figure 1/gel image/8.tif]

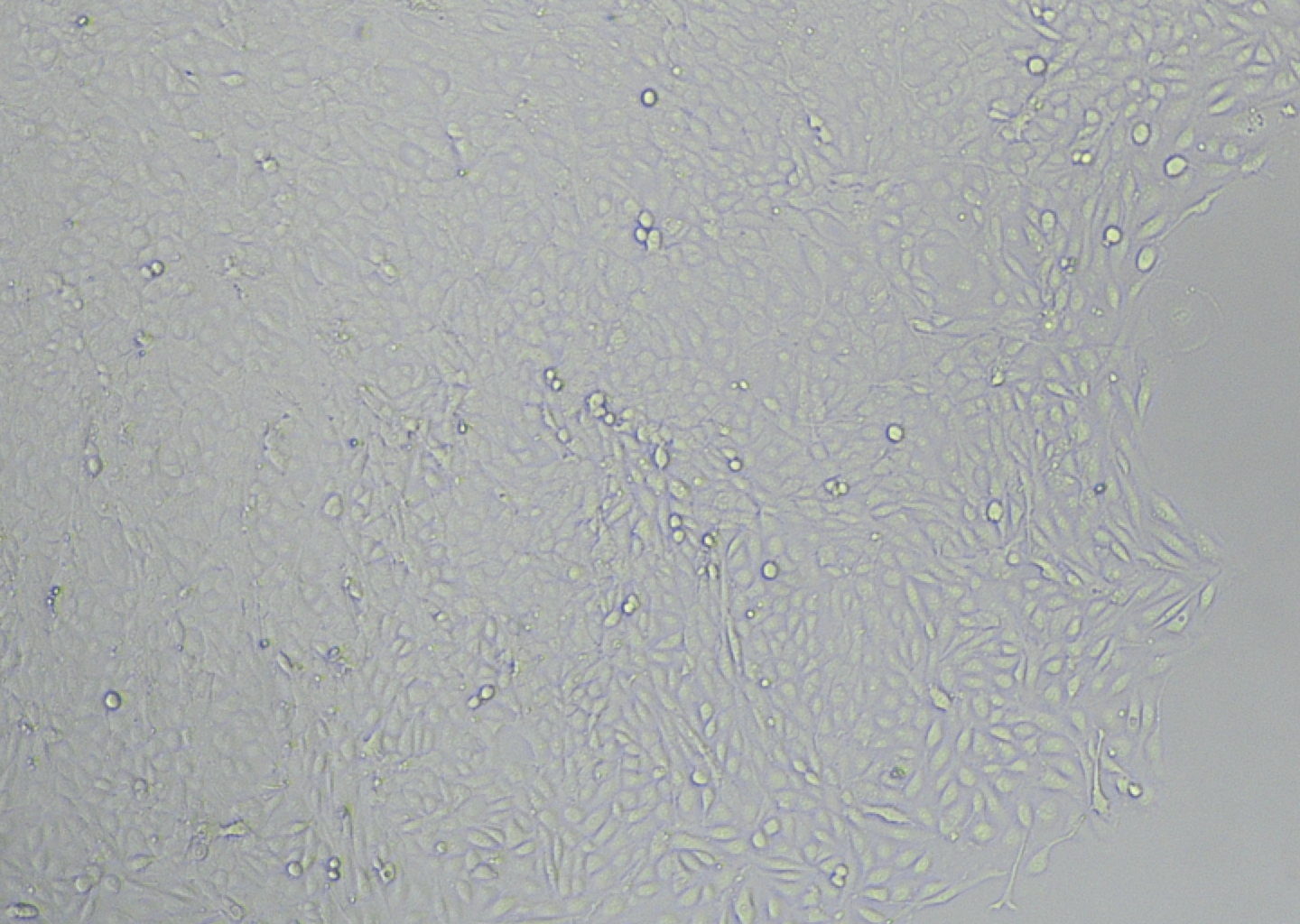

Supplement: Supplementary file 1 [file Data_Sheet_1.ZIP › Dr Jiang original data 1/figure 1/microscopy images/1.tif]

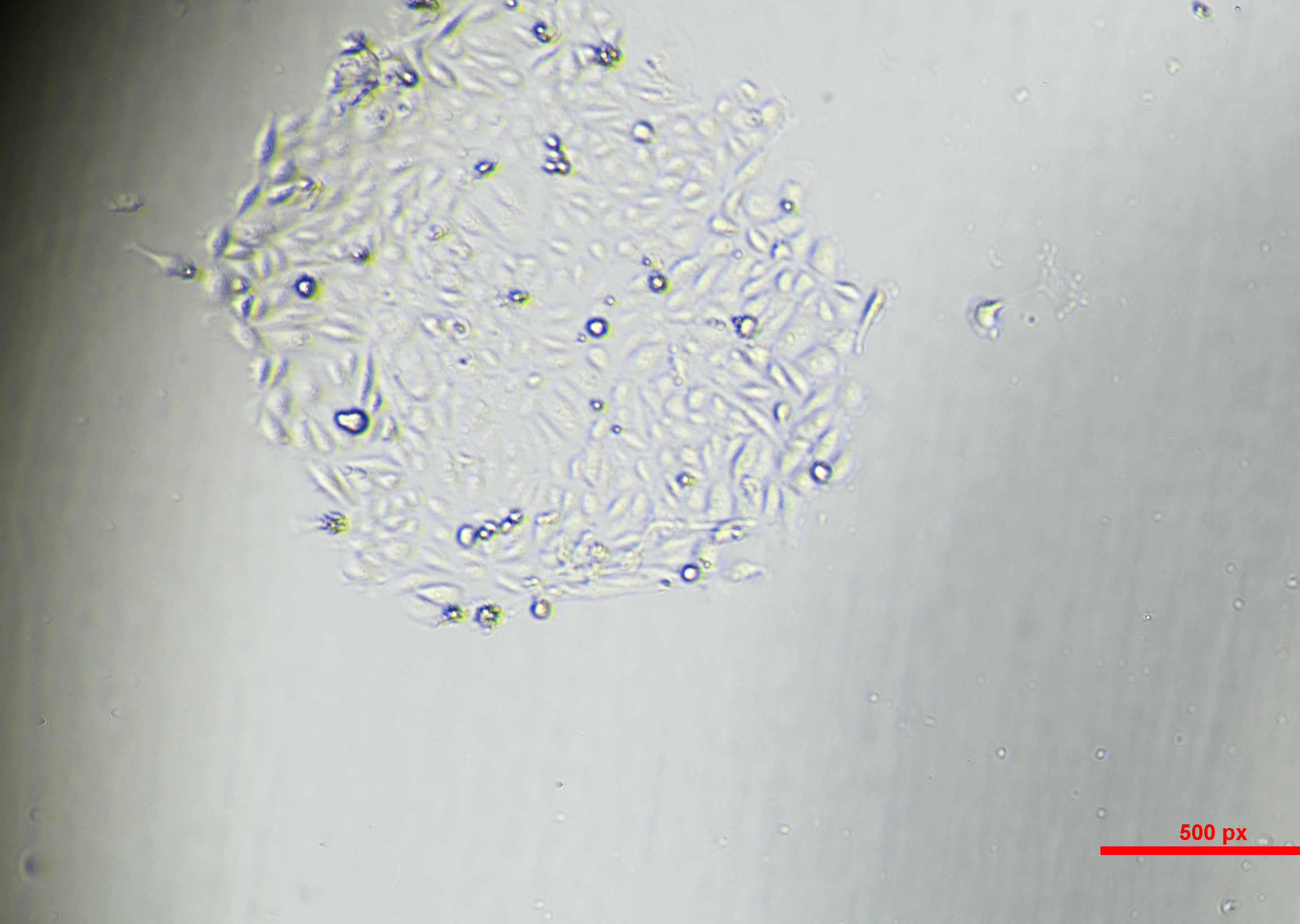

Supplement: Supplementary file 1 [file Data_Sheet_1.ZIP › Dr Jiang original data 1/figure 1/microscopy images/Captured27.tif]

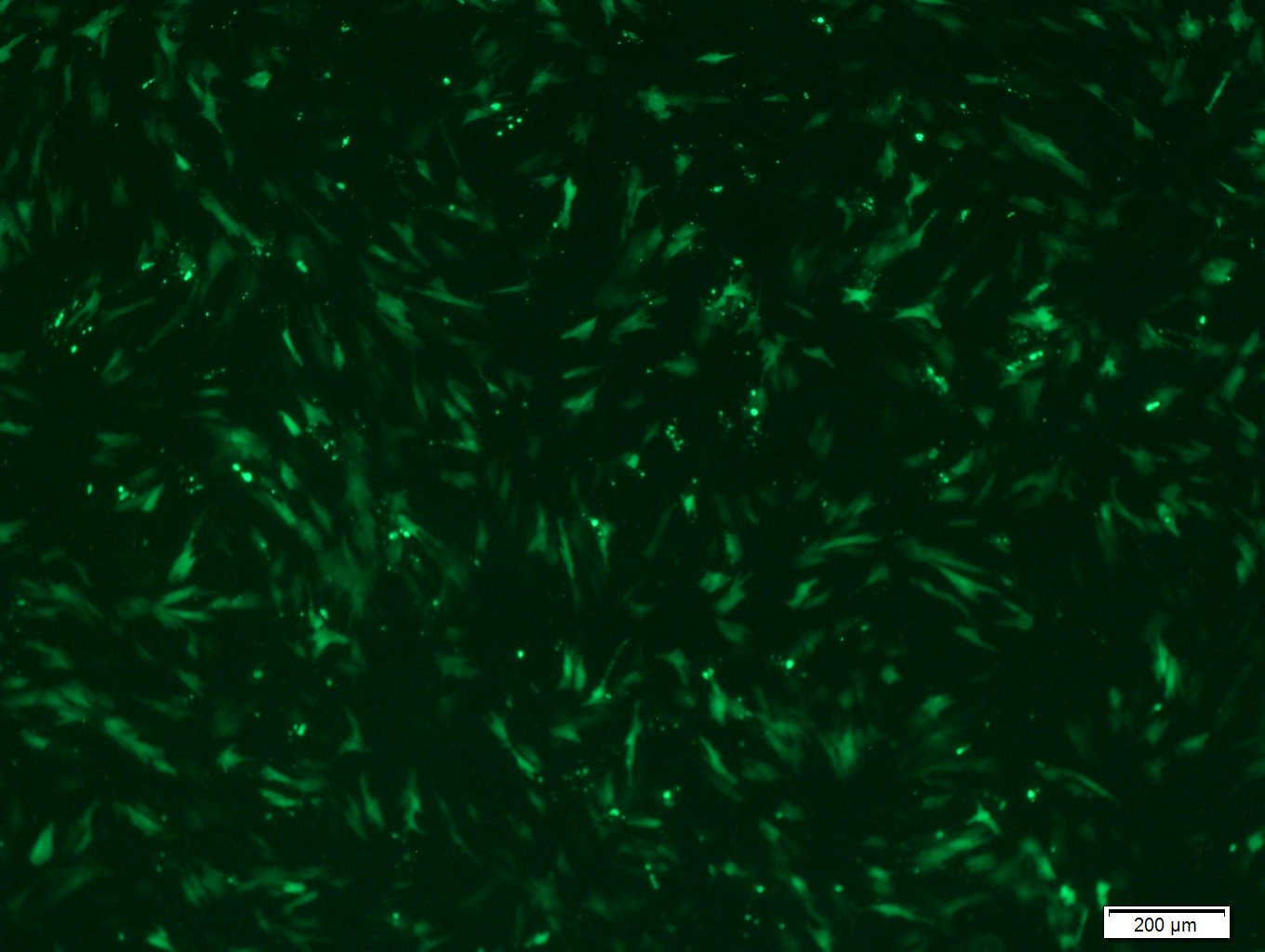

Supplement: Supplementary file 1 [file Data_Sheet_1.ZIP › Dr Jiang original data 1/figure 1/microscopy images/图像_9181.jpg]

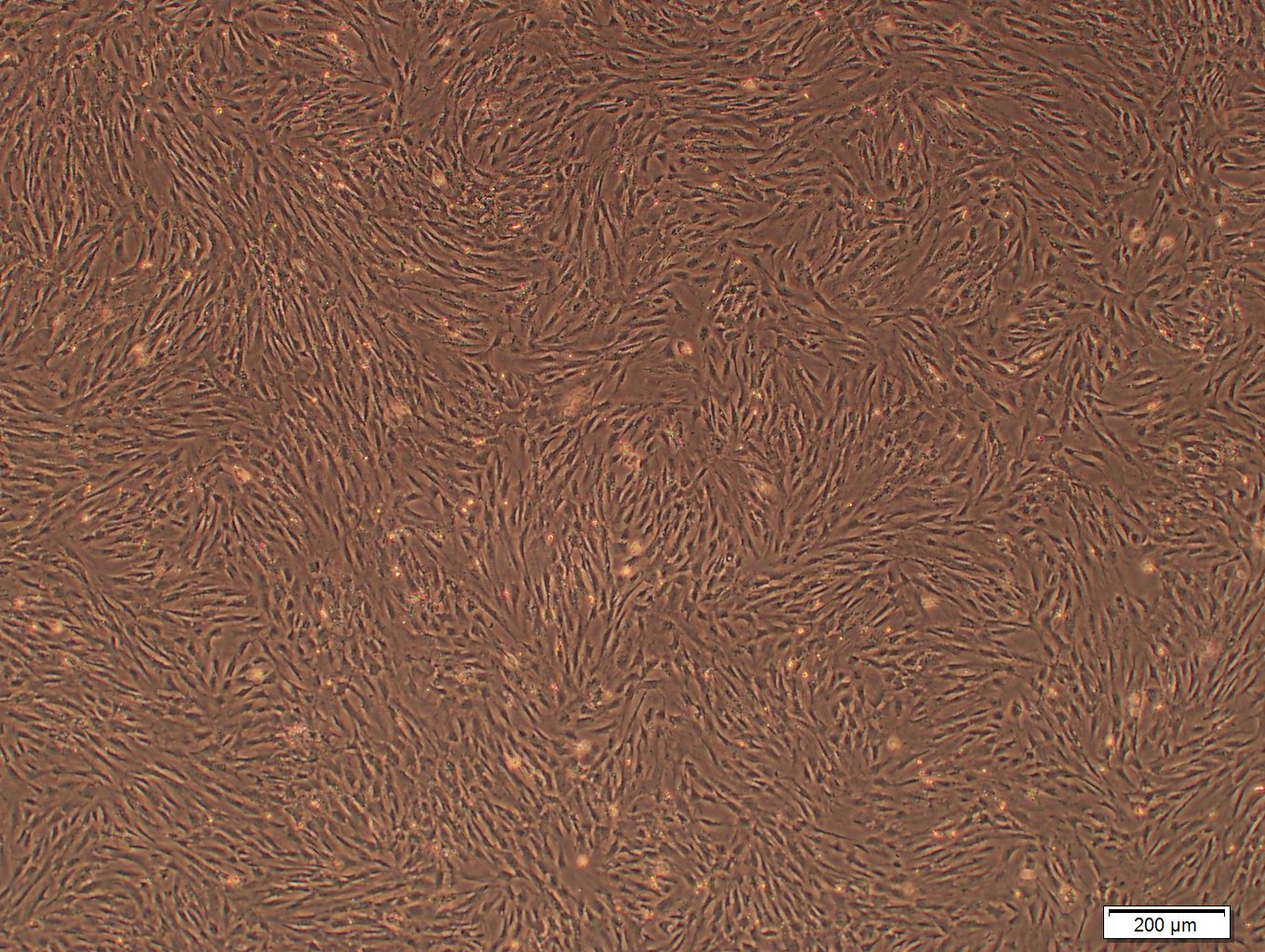

Supplement: Supplementary file 1 [file Data_Sheet_1.ZIP › Dr Jiang original data 1/figure 1/microscopy images/图像_9182.jpg]

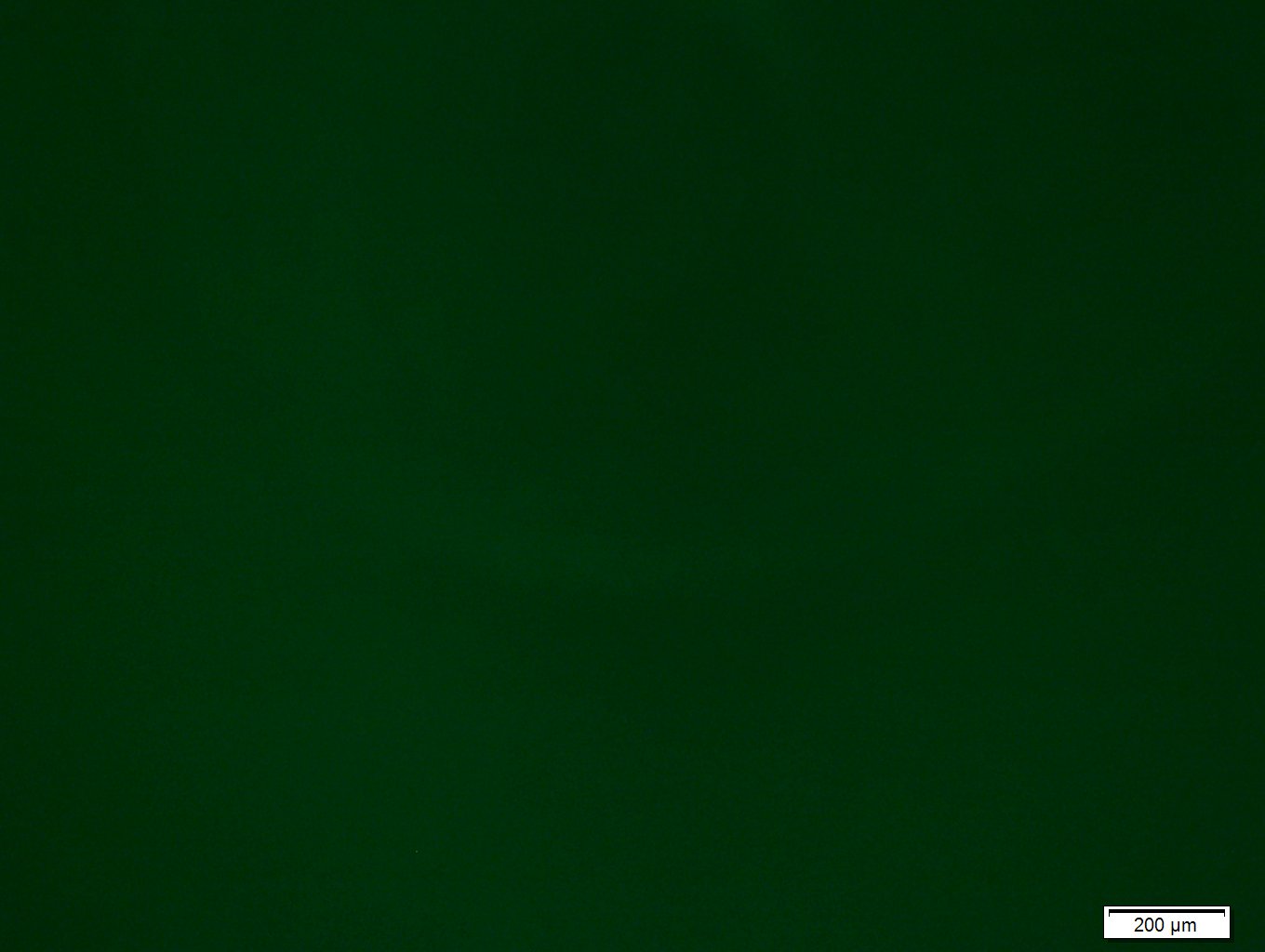

Supplement: Supplementary file 1 [file Data_Sheet_1.ZIP › Dr Jiang original data 1/figure 1/microscopy images/图像_9208.jpg]

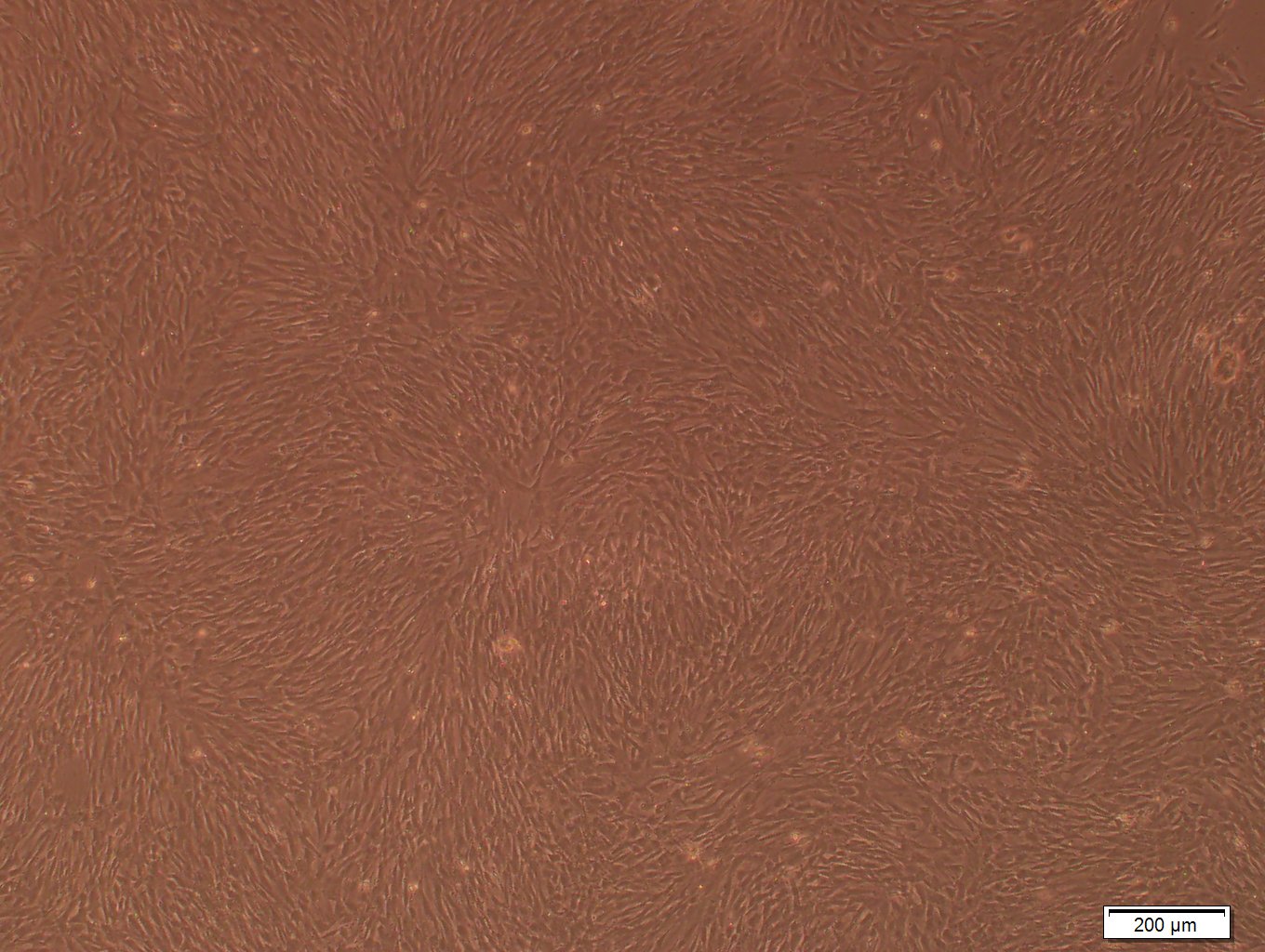

Supplement: Supplementary file 1 [file Data_Sheet_1.ZIP › Dr Jiang original data 1/figure 1/microscopy images/图像_9209.jpg]

Color Key

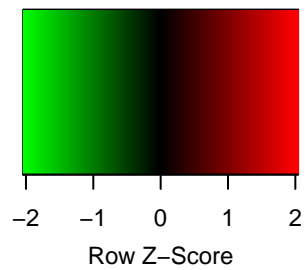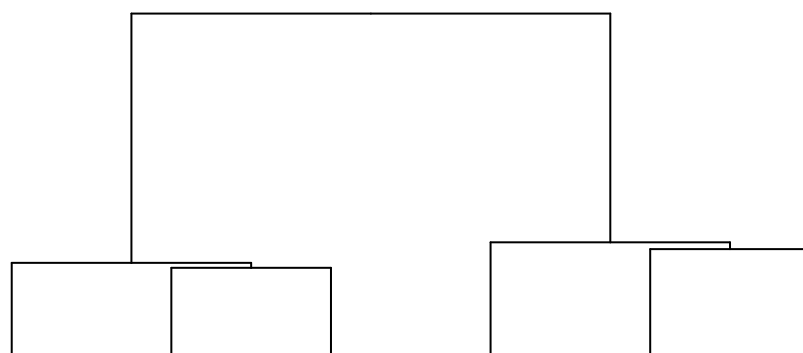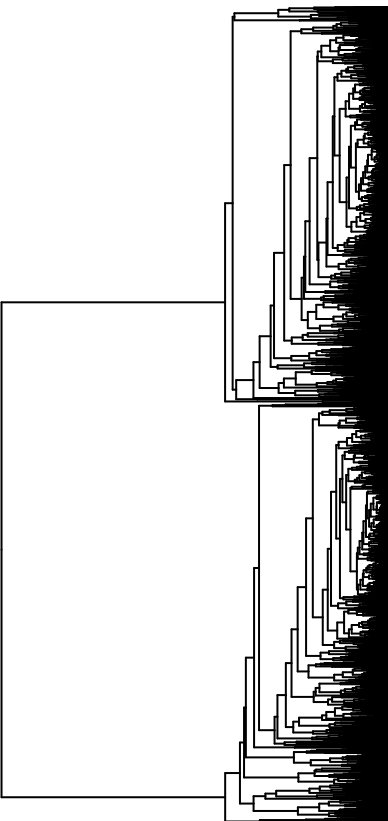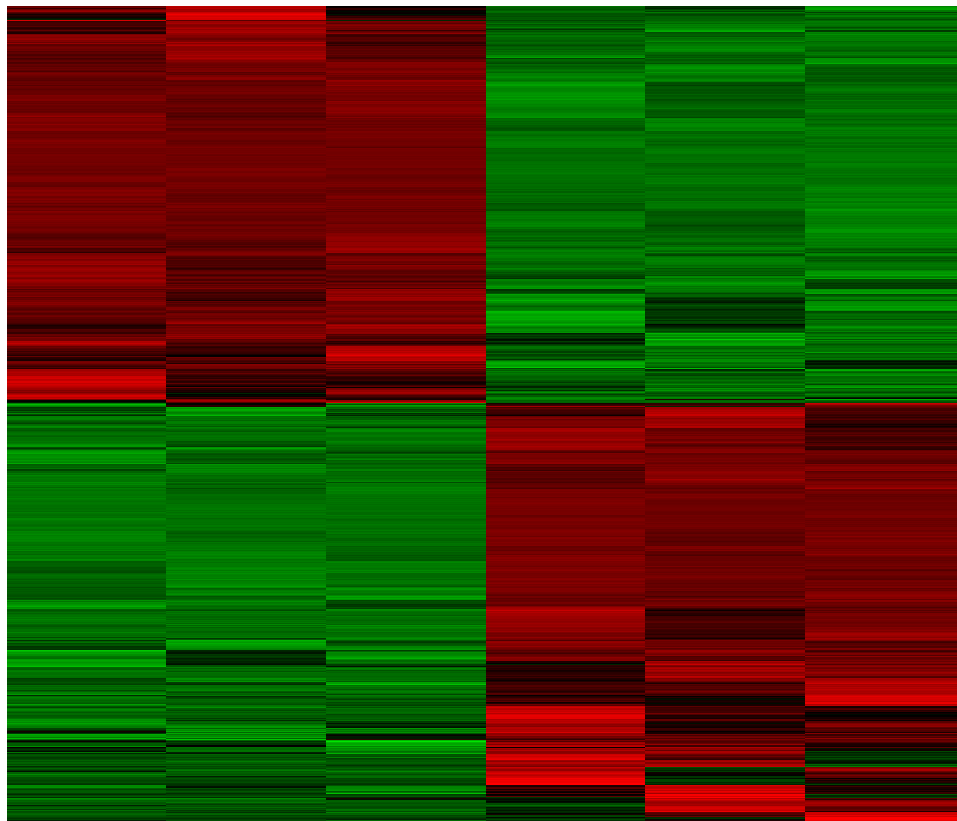

ACKOcell.3

ACKOcell.2

ACKOcell.1

MEC.1

MEC.2

MEC.3

Supplement: Supplementary file 1 [file Data_Sheet_1.ZIP › Dr Jiang original data 1/figure 3/ACKOvsMEC.cluster.pdf]

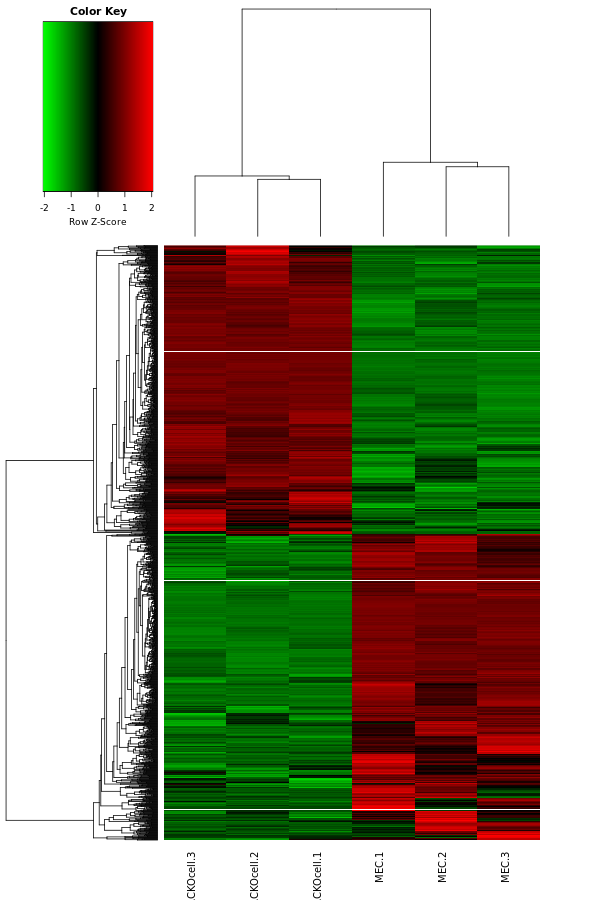

Supplement: Supplementary file 1 [file Data_Sheet_1.ZIP › Dr Jiang original data 1/figure 3/ACKOvsMEC.cluster.png]

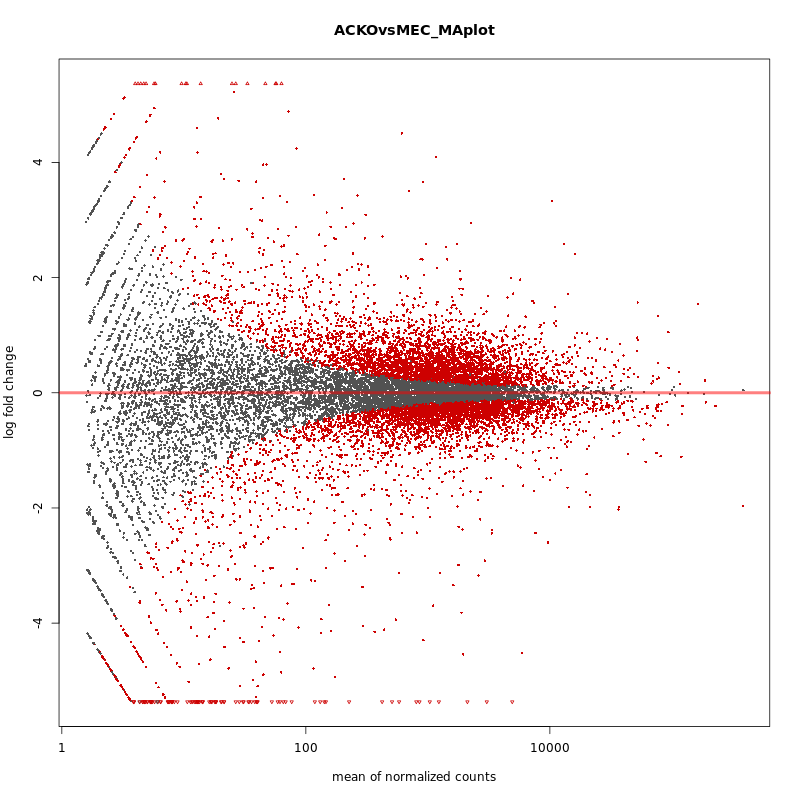

Supplement: Supplementary file 1 [file Data_Sheet_1.ZIP › Dr Jiang original data 1/figure 3/ACKOvsMEC.DESeq2.MAplot.png]

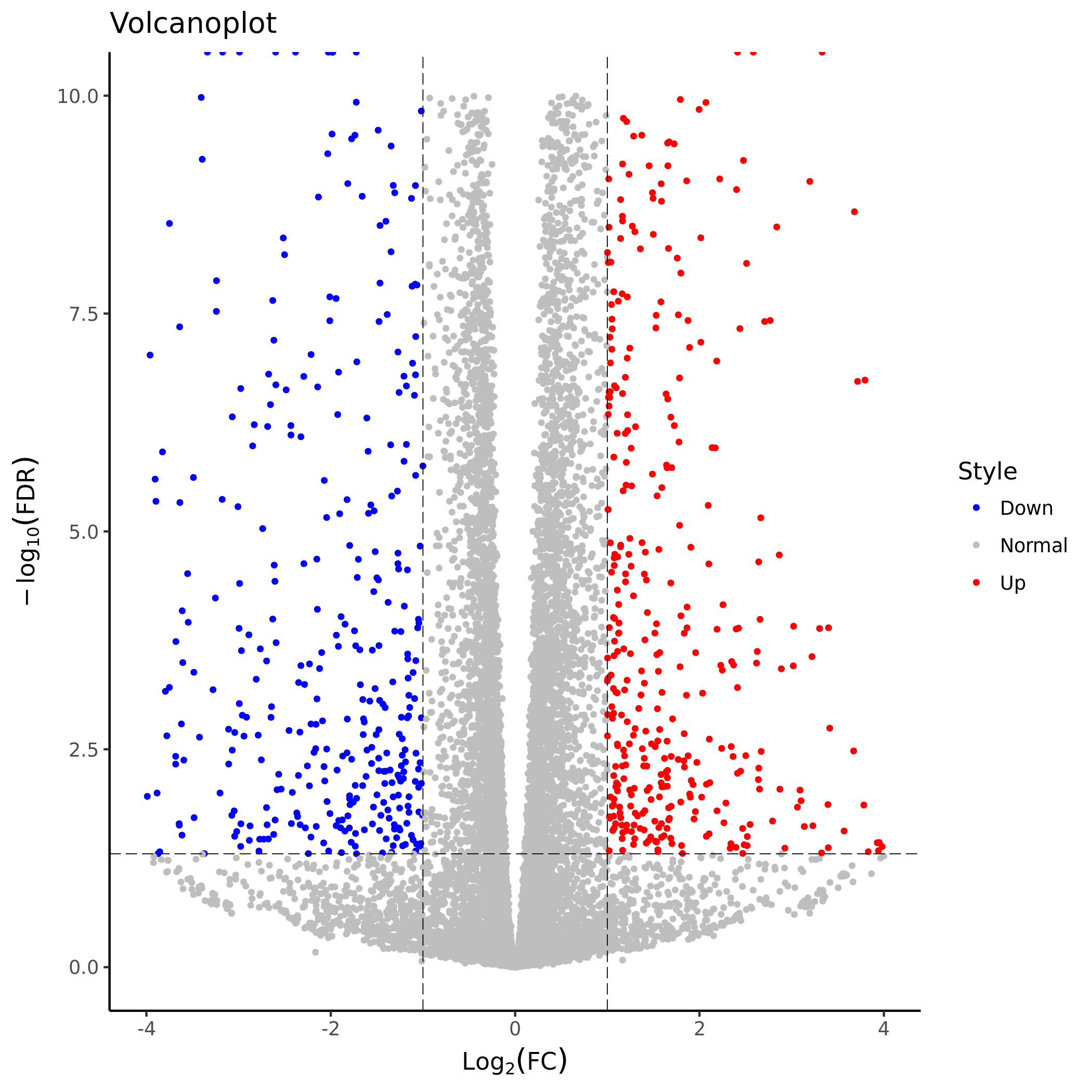

Supplement: Supplementary file 1 [file Data_Sheet_1.ZIP › Dr Jiang original data 1/figure 3/ACKOvsMEC.Volcano.png]

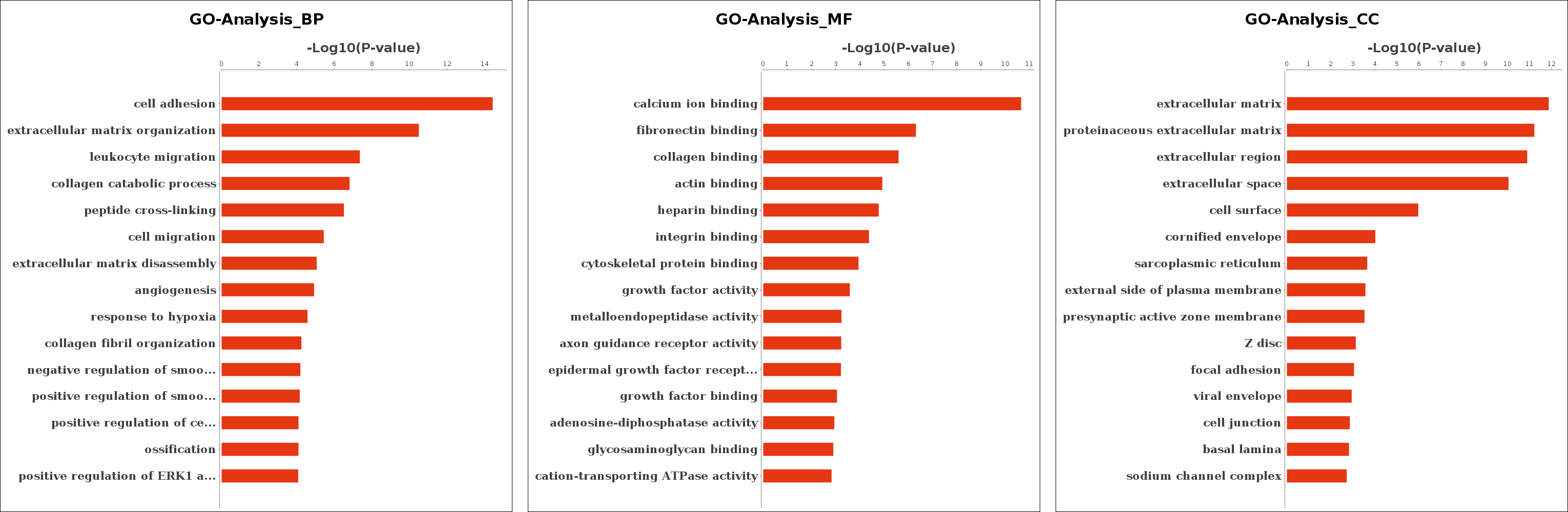

Supplement: Supplementary file 1 [file Data_Sheet_1.ZIP › Dr Jiang original data 1/figure 4/ACKOvsMEC.GO-Analysis-Log10P_All.png]

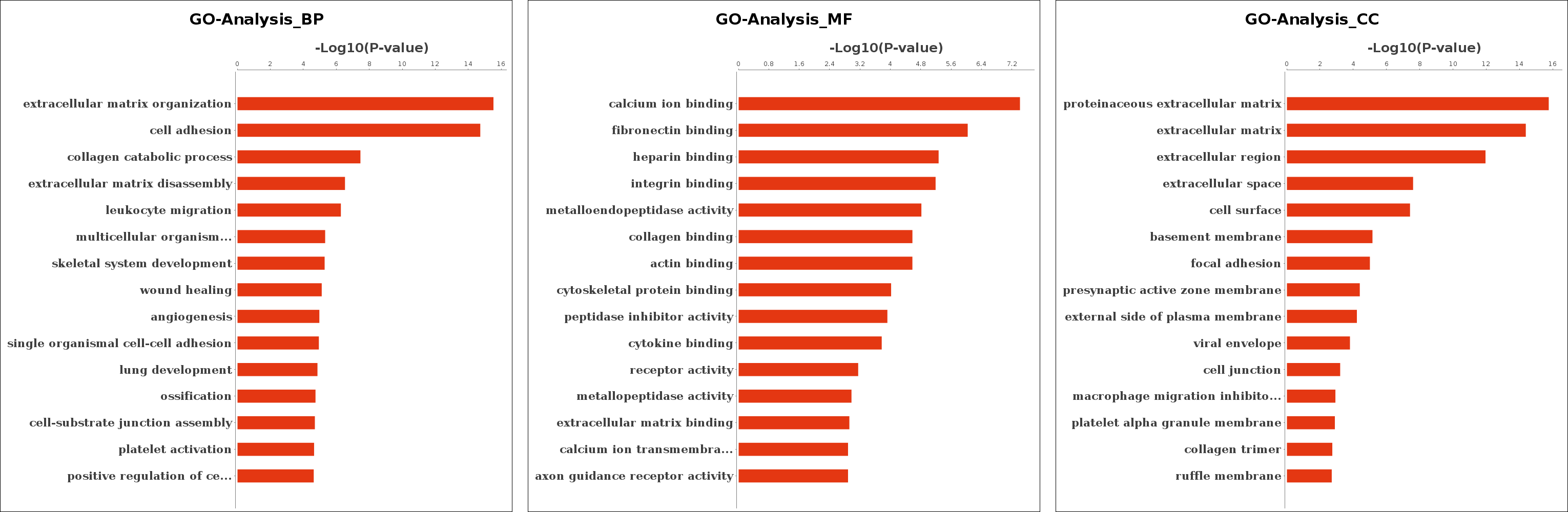

Supplement: Supplementary file 1 [file Data_Sheet_1.ZIP › Dr Jiang original data 1/figure 4/ACKOvsMEC.GO-Analysis-Log10P_Down.png]

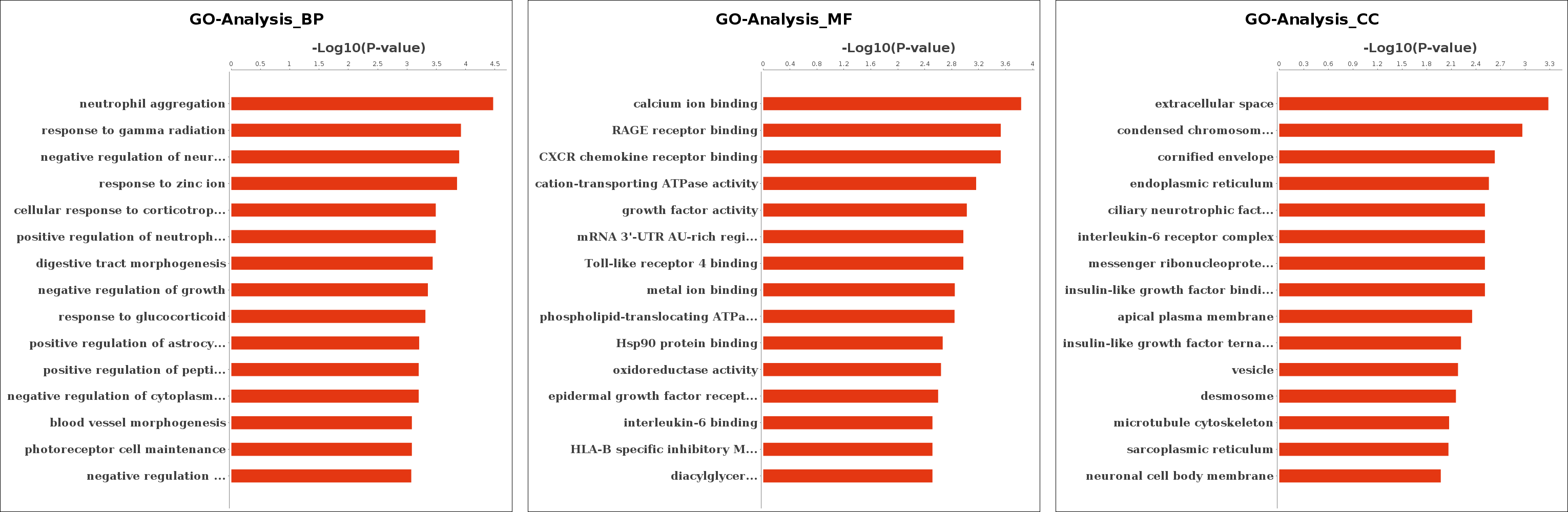

Supplement: Supplementary file 1 [file Data_Sheet_1.ZIP › Dr Jiang original data 1/figure 4/ACKOvsMEC.GO-Analysis-Log10P_Up.png]

Top 20 of GO enrichment

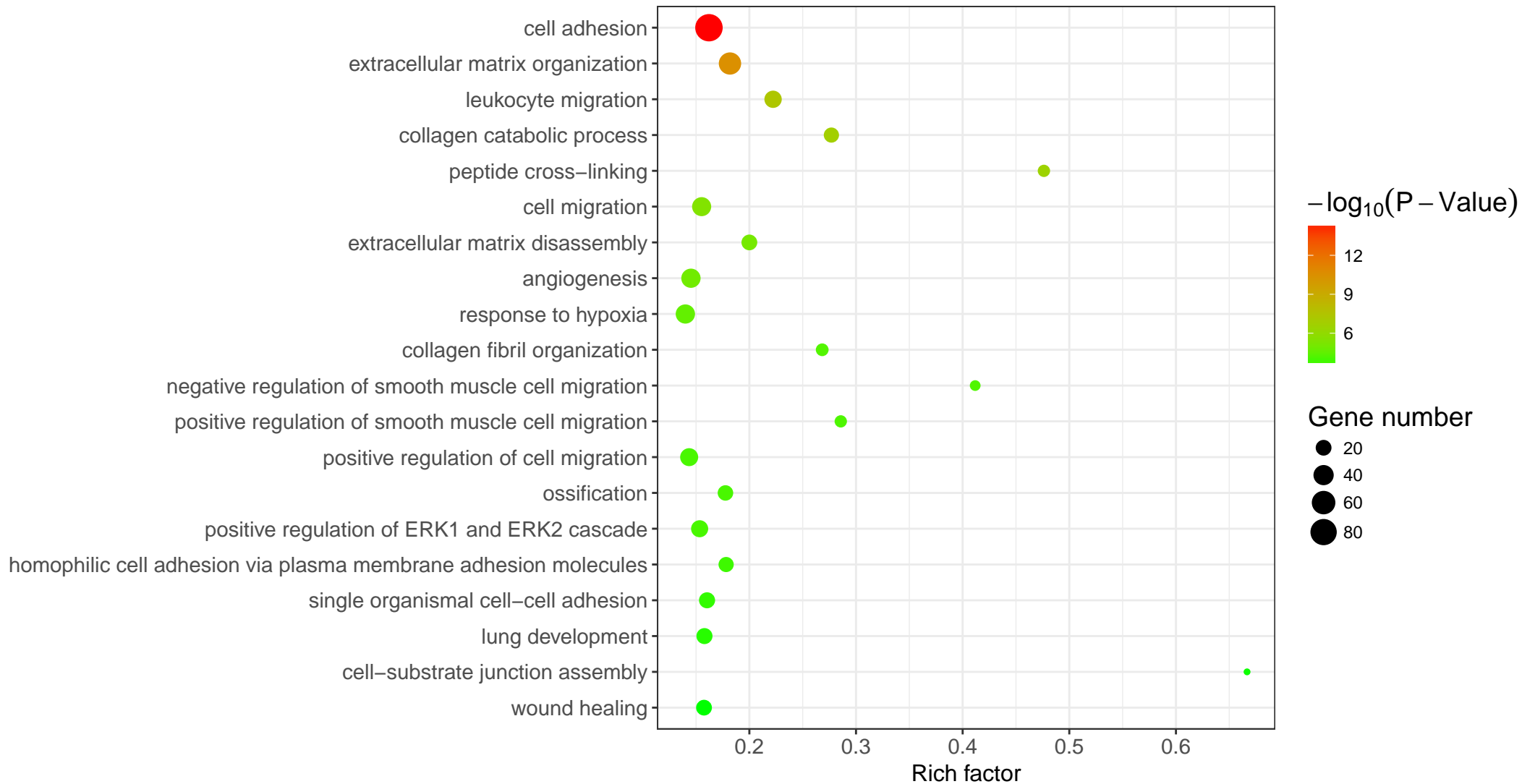

Supplement: Supplementary file 1 [file Data_Sheet_1.ZIP › Dr Jiang original data 1/figure 4/ACKOvsMEC_1_GO_Enrichment.pdf]

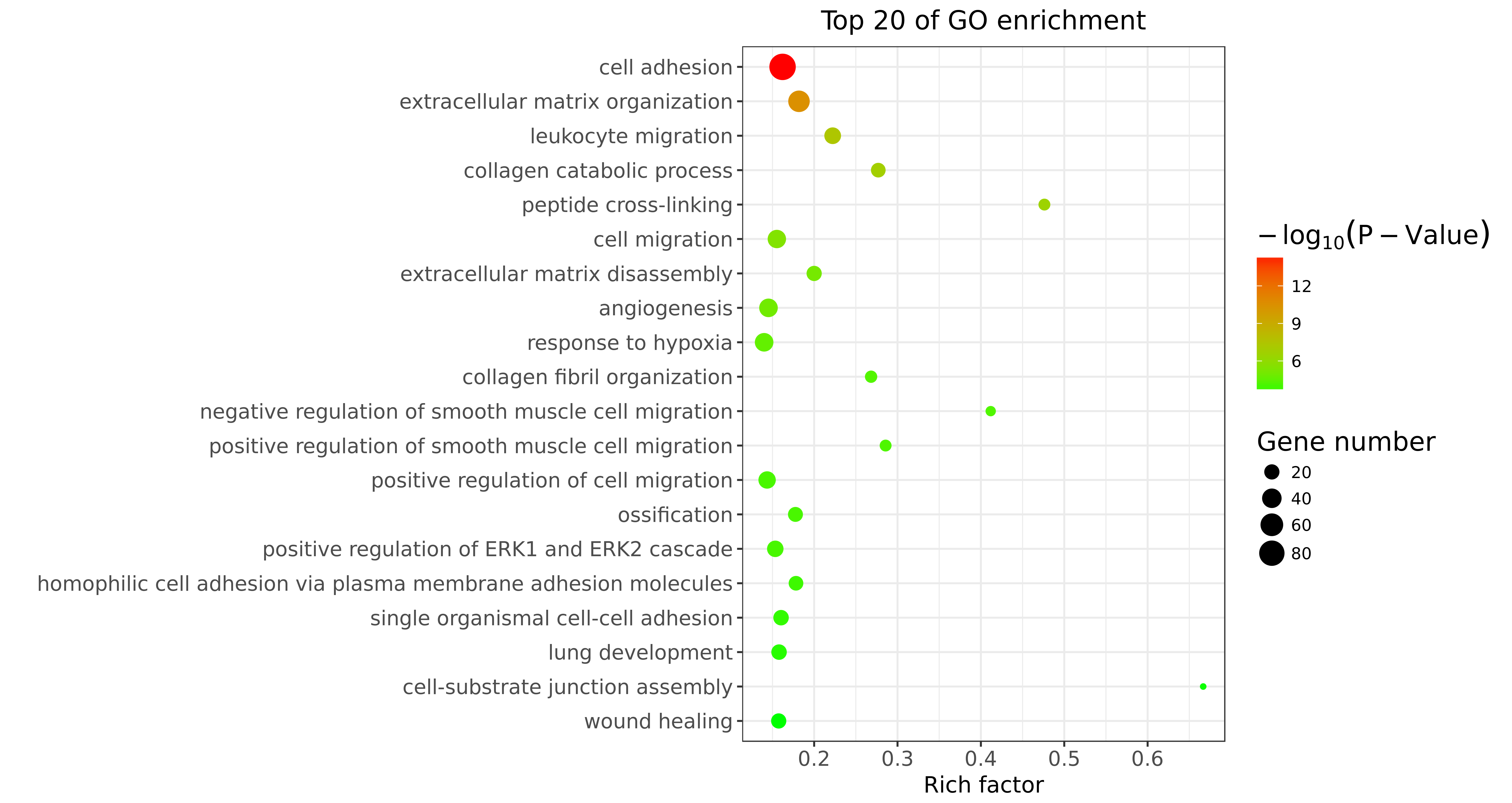

Supplement: Supplementary file 1 [file Data_Sheet_1.ZIP › Dr Jiang original data 1/figure 4/ACKOvsMEC_1_GO_Enrichment.png]

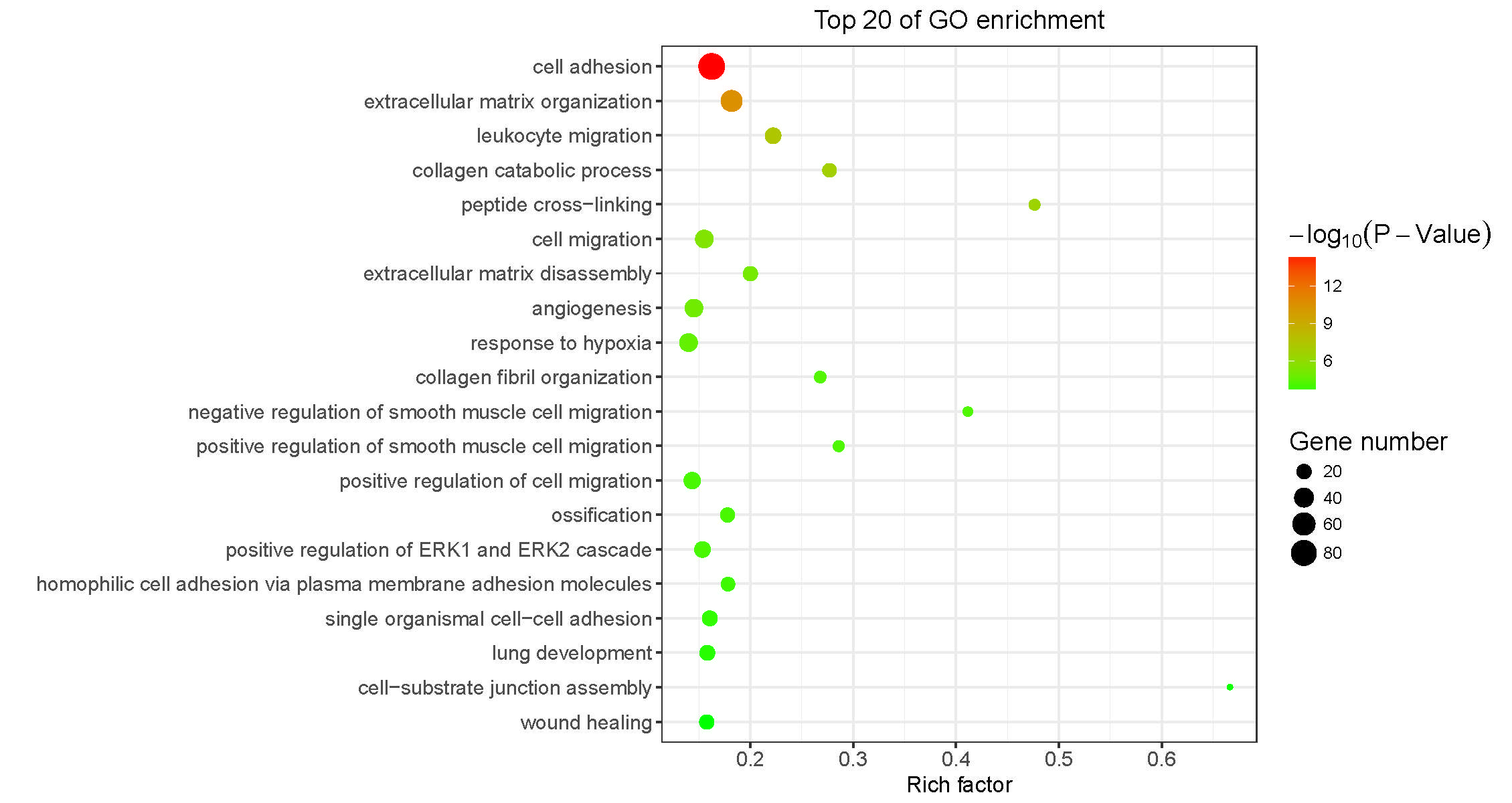

Supplement: Supplementary file 1 [file Data_Sheet_1.ZIP › Dr Jiang original data 1/figure 4/ACKOvsMEC_1_GO_Enrichment.tif]

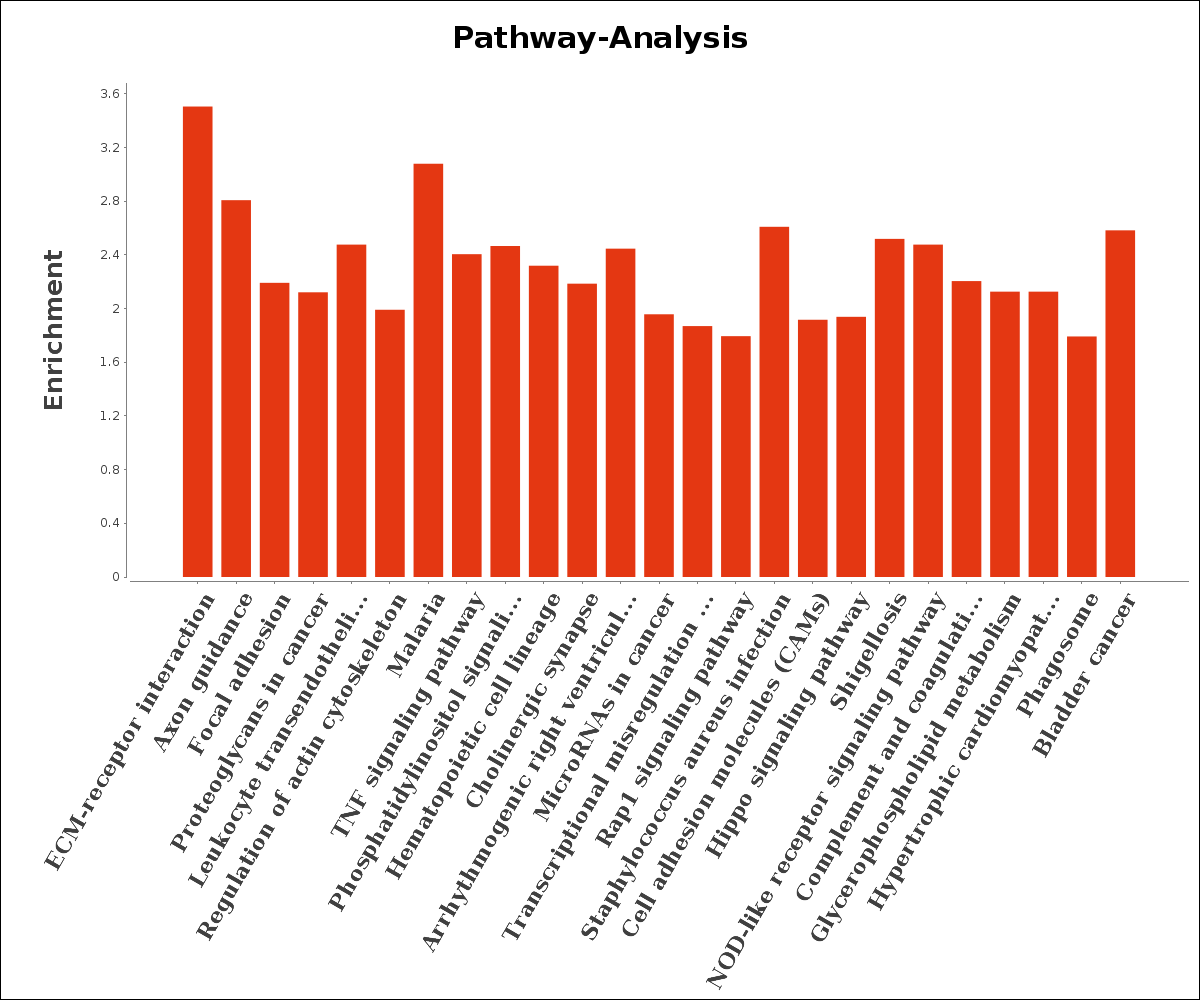

Supplement: Supplementary file 2 [file Data_Sheet_2.ZIP › Dr Jiang original data 2/figure 5/ACKOvsMEC.Path-Analysis-Enrichment.All.png]

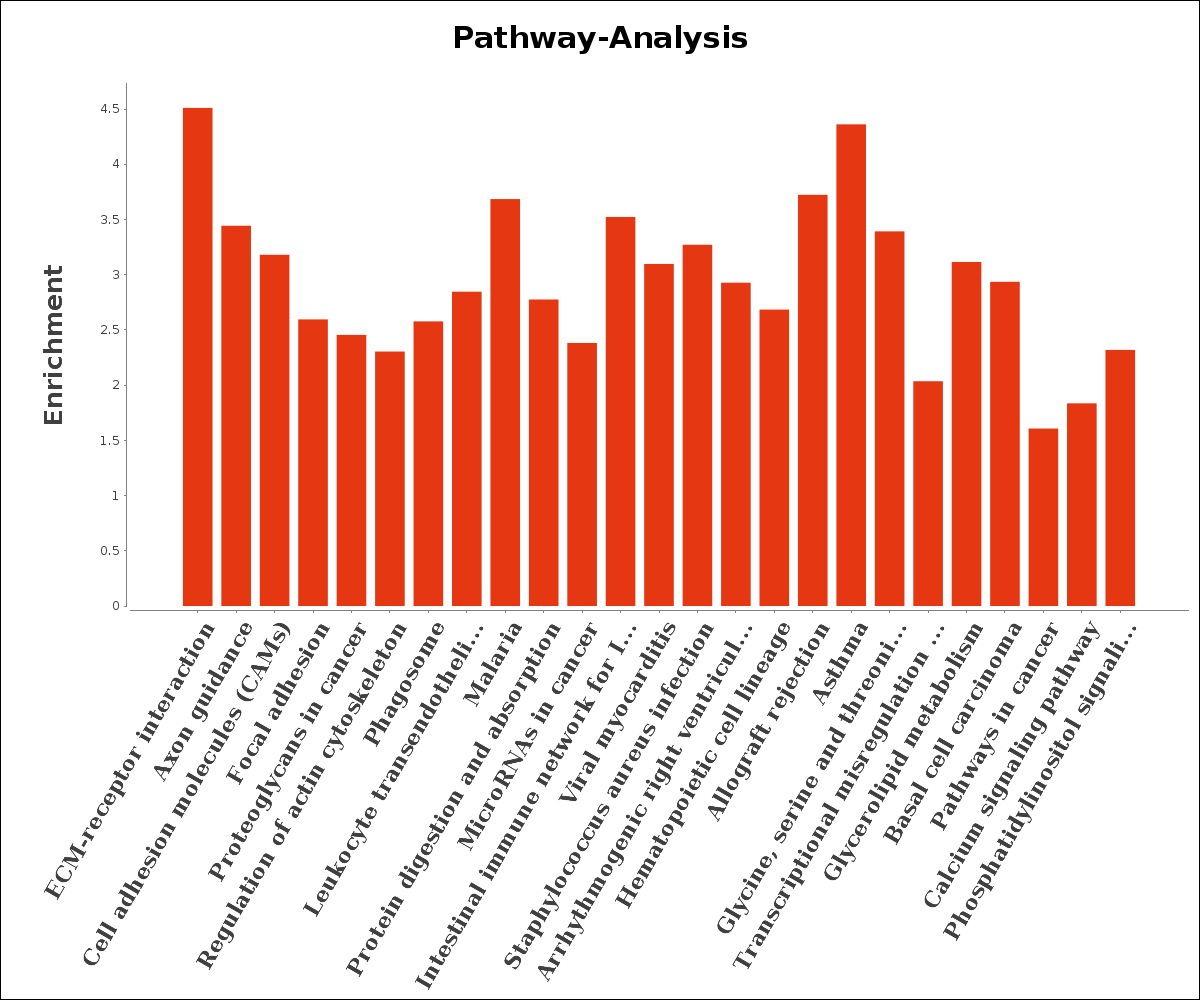

Supplement: Supplementary file 2 [file Data_Sheet_2.ZIP › Dr Jiang original data 2/figure 5/ACKOvsMEC.Path-Analysis-Enrichment.Down.png]

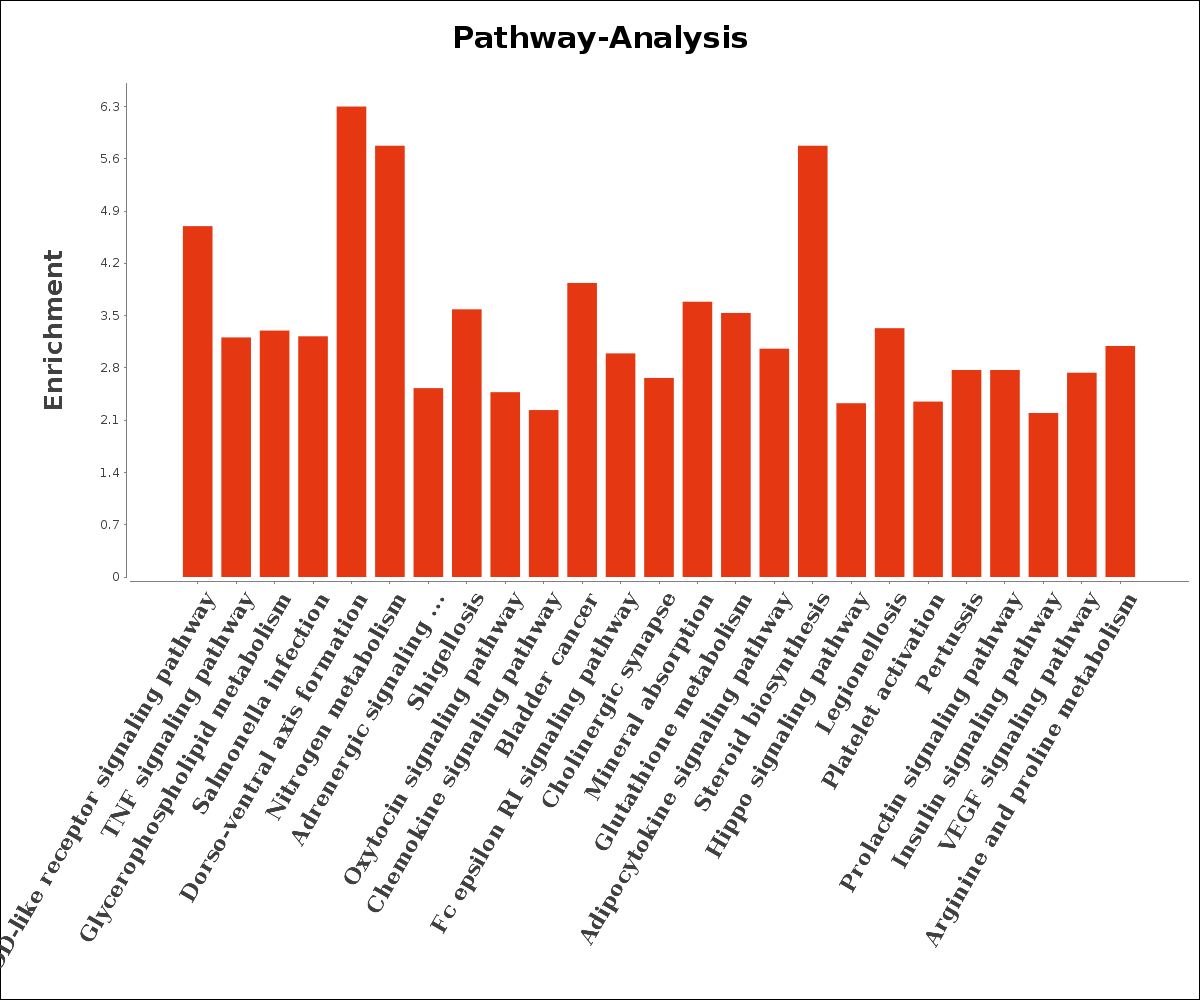

Supplement: Supplementary file 2 [file Data_Sheet_2.ZIP › Dr Jiang original data 2/figure 5/ACKOvsMEC.Path-Analysis-Enrichment.Up.png]

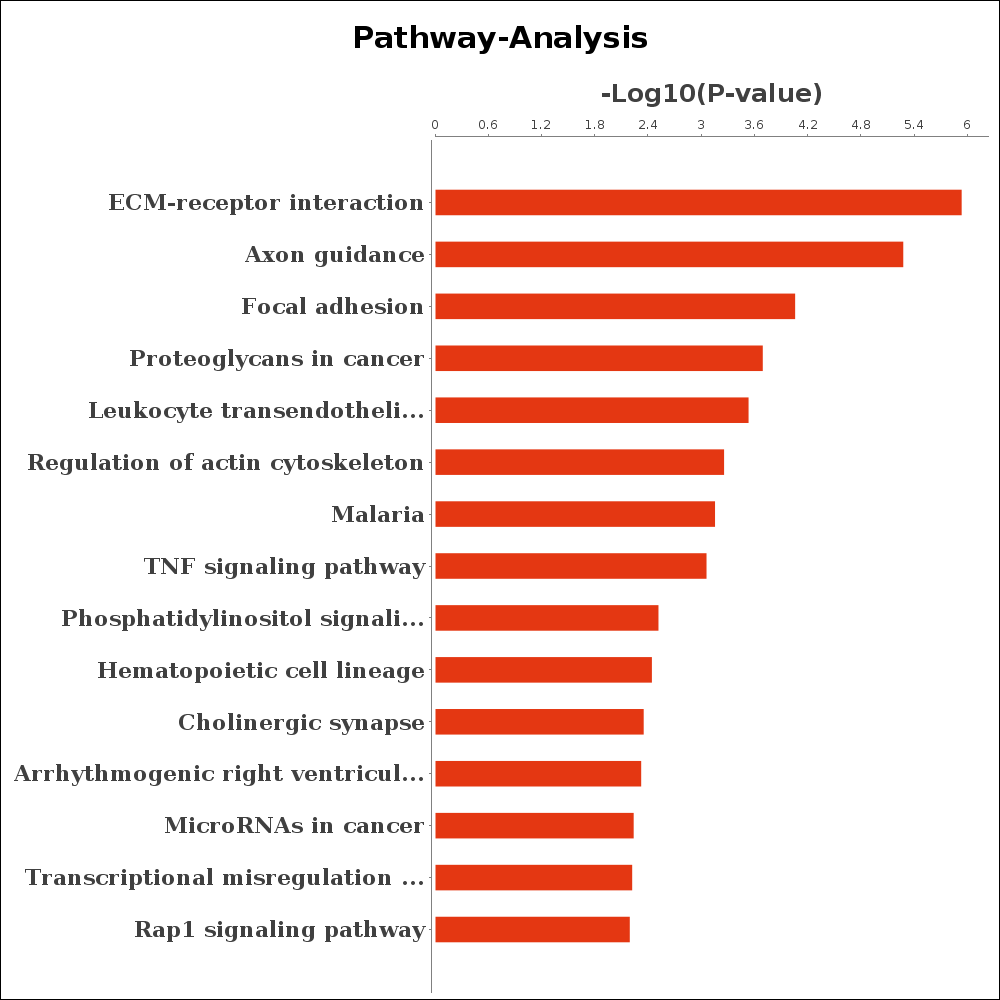

Supplement: Supplementary file 2 [file Data_Sheet_2.ZIP › Dr Jiang original data 2/figure 5/ACKOvsMEC.Path-Analysis-Log10P.All.png]

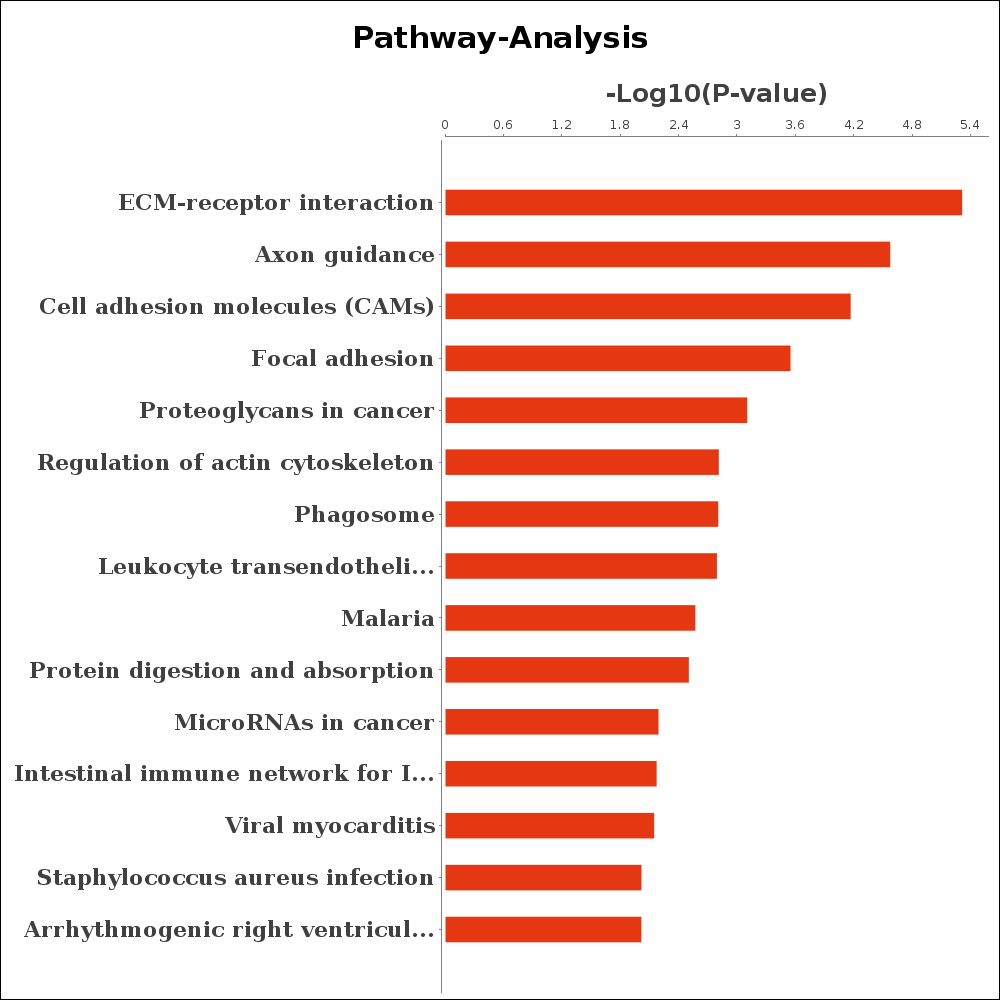

Supplement: Supplementary file 2 [file Data_Sheet_2.ZIP › Dr Jiang original data 2/figure 5/ACKOvsMEC.Path-Analysis-Log10P.Down.png]

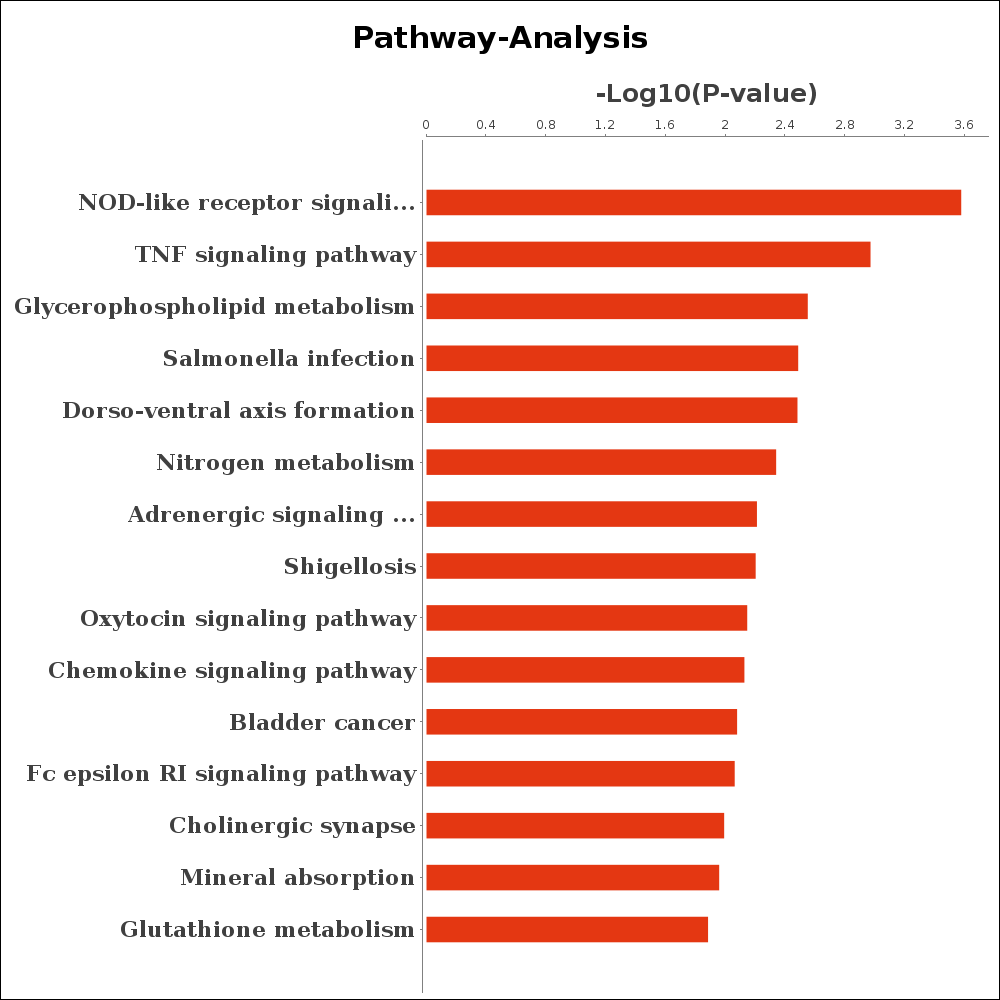

Supplement: Supplementary file 2 [file Data_Sheet_2.ZIP › Dr Jiang original data 2/figure 5/ACKOvsMEC.Path-Analysis-Log10P.Up.png]

Top 20 of Pathway enrichment

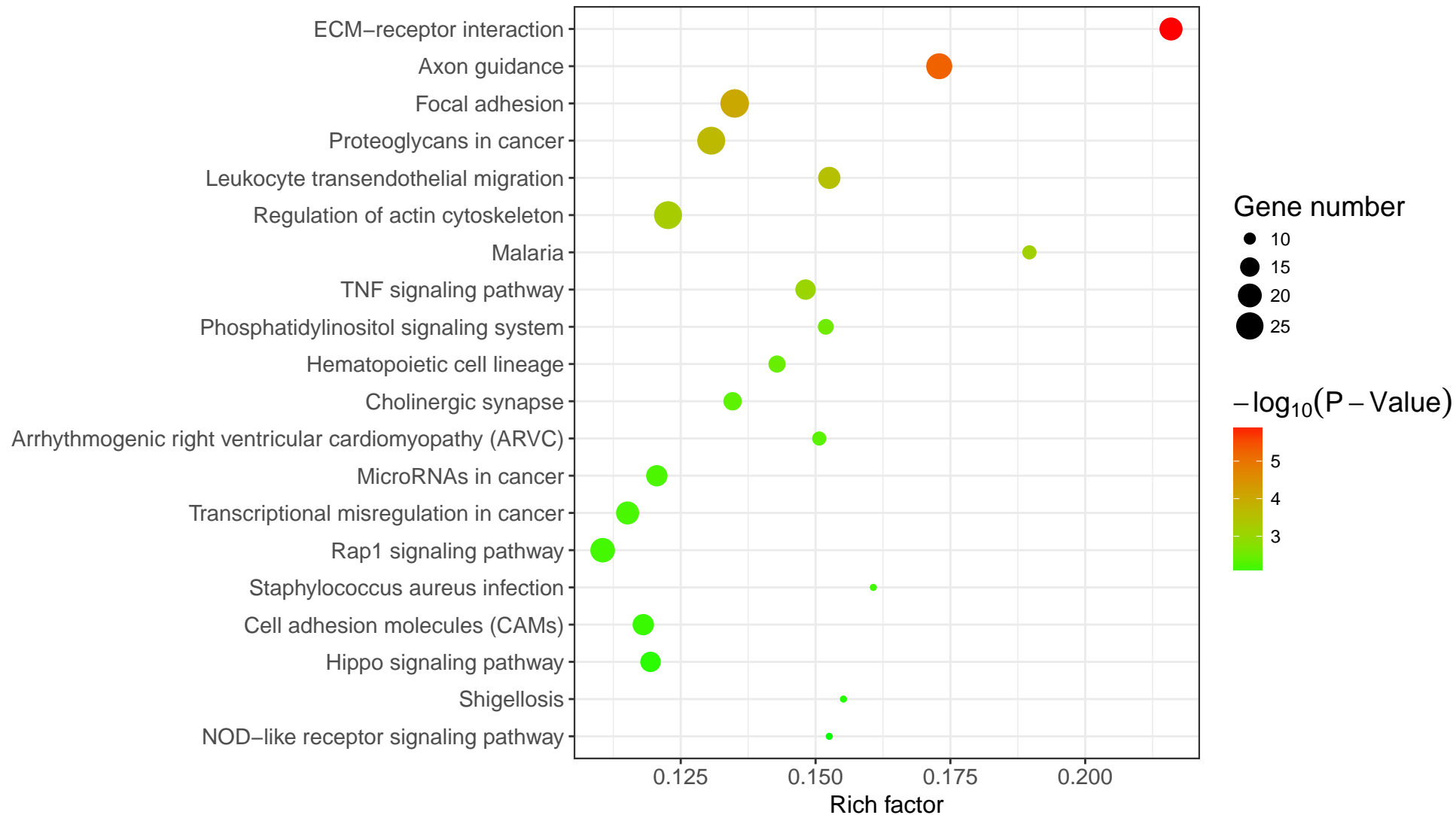

Supplement: Supplementary file 2 [file Data_Sheet_2.ZIP › Dr Jiang original data 2/figure 5/ACKOvsMEC_1_Pathway_Enrichment.pdf]

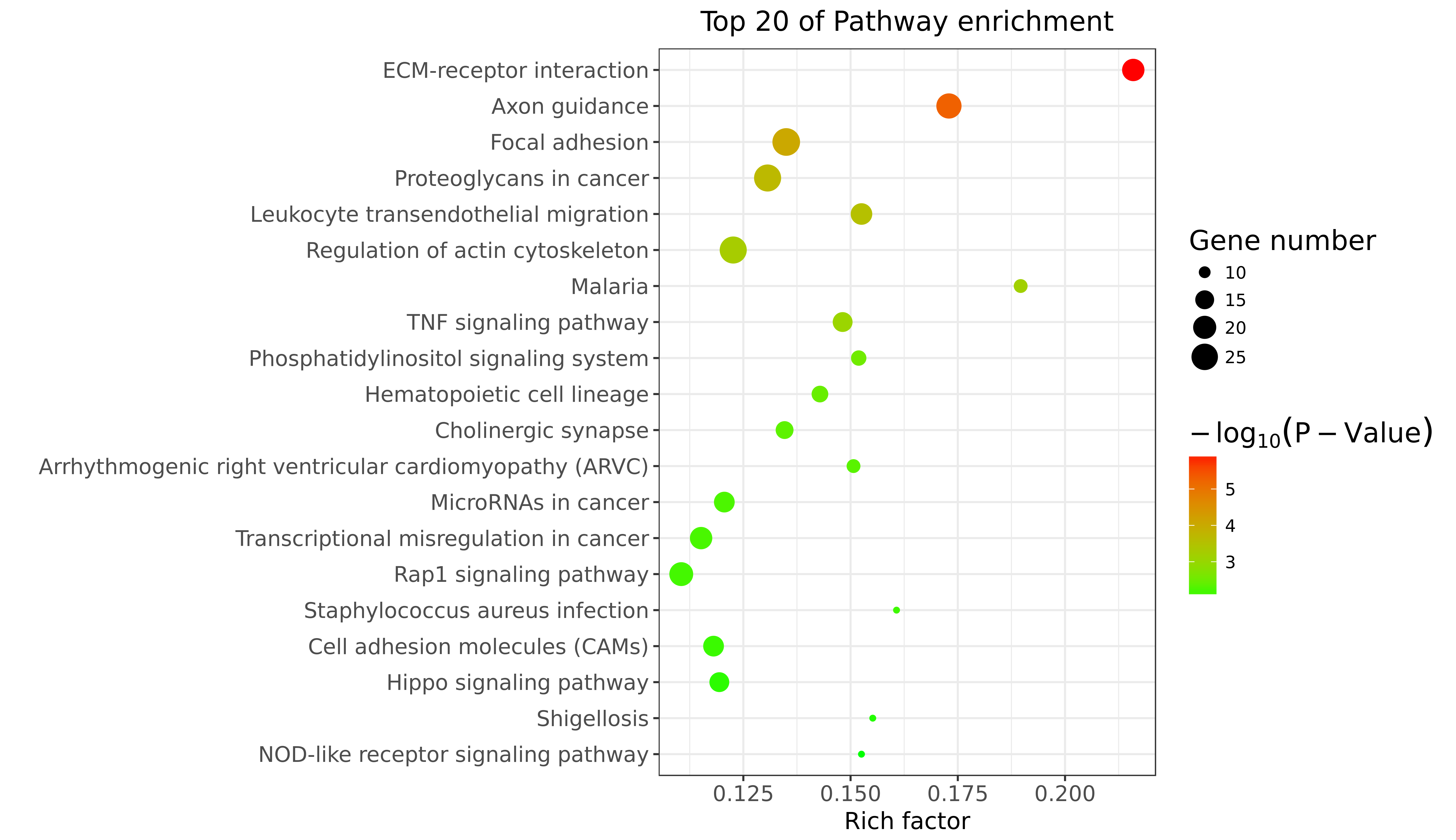

Supplement: Supplementary file 2 [file Data_Sheet_2.ZIP › Dr Jiang original data 2/figure 5/ACKOvsMEC_1_Pathway_Enrichment.png]

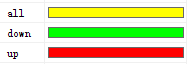

Supplement: Supplementary file 2 [file Data_Sheet_2.ZIP › Dr Jiang original data 2/figure 6/ColorBar.png]

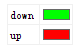

Supplement: Supplementary file 2 [file Data_Sheet_2.ZIP › Dr Jiang original data 2/figure 7/ColorBar.png]
